# The yapsin family of aspartyl proteases regulate glucose homeostasis in *Candida glabrata*

**DOI:** 10.1016/j.jbc.2022.101593

**Published:** 2022-01-17

**Authors:** Fizza Askari, Mubashshir Rasheed, Rupinder Kaur

**Affiliations:** 1 Laboratory of Fungal Pathogenesis, Centre for DNA Fingerprinting and Diagnostics, Hyderabad, India; 2 Graduate Studies, Regional Centre for Biotechnology, Faridabad, Haryana, India

**Keywords:** aspartic protease, glucose, respiration, proteomics, human pathogenic fungi, GPI-linked aspartyl proteases, glucose sensing and uptake, hexose transporters, membrane proteome, high-affinity glucose sensor Snf3, COX, cytochrome C oxidase, ETP, electron transport, GO, Gene Ontology, GPI, glycosylphosphatidylinositol, TMF, total membrane fraction

## Abstract

Invasive candidiasis poses a major healthcare threat. The human opportunistic fungal pathogen *Candida glabrata*, which causes mucosal and deep-seated infections, is armed with distinct virulence attributes, including a family of 11 glycosylphosphatidylinositol-linked aspartyl proteases, CgYapsins. Here, we have profiled total membrane proteomes of the *C. glabrata wildtype* and 11 proteases-deficient strain, *Cgyps1-11Δ*, by mass spectrometry analysis and uncovered a novel role for fungal yapsins in glucose sensing and homeostasis. Furthermore, through label-free quantitative membrane proteome analysis, we showed differential abundance of 42% of identified membrane proteins, with electron transport chain and glycolysis proteins displaying lower and higher abundance in *Cgyps1-11Δ* cells, compared with *wildtype* cells, respectively. We also demonstrated elevated glucose uptake and upregulation of genes that code for the low-glucose sensor CgSnf3, transcriptional regulators CgMig1 and CgRgt1, and hexose transporter CgHxt2/10 in the *Cgyps1-11Δ* mutant. We further elucidated a potential underlying mechanism through genetic and transcript measurement analysis under low- and high-glucose conditions and found *CgSNF3* deletion to rescue high glucose uptake and attenuated growth of the *Cgyps1-11Δ* mutant in YPD medium, thereby linking CgYapsins with regulation of the CgSnf3-dependent low-glucose sensing pathway. Last, high ethanol production, diminished mitochondrial membrane potential, and elevated susceptibility to oxidative phosphorylation inhibitors point toward increased fermentative and decreased respiratory metabolism in the *Cgyps1-11Δ* mutant. Altogether, our findings revealed new possible glucose metabolism-regulatory roles for putative cell surface-associated CgYapsins and advanced our understanding of fungal carbohydrate homeostasis mechanisms.

Extracellular aspartyl proteases are key virulence factors in pathogenic fungi, as these play a critical role in host invasion and infection establishment (1, 2, 3). Based on their localization, fungal extracellular aspartyl proteases have been divided into two classes: secretory and glycosylphosphatidylinositol (GPI) linked, with the latter class possessing the GPI-anchor sequence motif in their C termini, which facilitates attachment to the cell wall and/or the cell membrane (1, 2, 3, 4). The secretory aspartyl proteases in human fungal pathogens have been associated with degradation of a wide spectrum of host proteins including E-cadherin, cathepsin D, mucin, clotting and complement factors, and extracellular matrix proteins, whereas the GPI-anchored aspartyl proteases have been implicated in fungal cell wall organization, processing of the fungal cell wall proteins, and modulating the adherence and damage to host cells as well as suppressing the host immune response (1, 2, 3, 5, 6).

*Candida* species are the most common fungal pathogens that cause invasive infections in immunocompromised human hosts, with these infections associated with high economic burden, morbidity, and mortality (7, 8). Five *Candida* species, *Candida albicans*, *Candida glabrata*, *Candida tropicalis*, *Candida parapsilosis*, and *Candida krusei*, have been reported to contribute to more than 90% of *Candida* bloodstream infections (7, 9). *C. glabrata* is a haploid nondimorphic yeast that grows by budding (6). Depending upon the geographical region, *C. glabrata* is the second to fourth most frequently isolated *Candida* species from the blood (9, 10, 11, 12). It also causes superficial mucosal infections and has been associated with high resistance toward azole and echinocandin antifungal drugs (6, 12).

An increased prevalence of *C. glabrata* has been reported in diabetic patients, with elevated glucose levels associated with *Candida* overgrowth (13, 14, 15). The capability of *C. glabrata* to proliferate in host macrophages *in vitro* is dependent upon reconfiguration of the carbon metabolism, reorganization of the chromatin structure and induction of pexophagy and autophagy in yeast cells, and blocking the acidification of the phagolysosome and activation of the NLRP3 inflammasome-dependent secretion of the proinflammatory cytokine IL-1β in macrophages (5, 16, 17, 18, 19). Of note, glucose homeostasis has recently been reported to play an important role in inflammasome activation and macrophage survival in response to *C. albicans* infections (20, 21). However, extracellular glucose sensing and transport mechanisms in *C. glabrata* and their contribution to pathogenesis (22) are yet to be fully elucidated.

A family of 11 putative GPI-linked aspartyl proteases, which are commonly referred to as CgYapsins, is a major contributor to the pathogenesis of *C. glabrata* (5, 6). A set of 11 genes, *CgYPS1–11*, which reside on three different chromosomes, Chromosome A (*CgYPS7*), E (*CgYPS2*, *YPS3–6*, and *YPS8–11*), and M (*CgYPS1*), code for CgYapsins, with *CgYPS3–6* and *CgYPS8–11* genes forming a cluster of eight genes on chromosome E (5). A central role for CgYapsins in virulence has been attributed to their functions in maintenance of the cell wall structure and composition, vacuole, pH and energy homeostasis, stress survival, regulation of the secretome, suppression of the macrophage proinflammatory immune response, and colonization of the mice and fly hosts (19, 23, 24, 25, 26, 27). Using mutants lacking single or multiple CgYapsin-encoding genes, it has been shown that CgYps1–11 aspartyl proteases are essential for biofilm formation and survival of *C. glabrata* in human and murine macrophages and a mouse model of systemic candidiasis (5, 19).

Furthermore, although CgYapsins are thought to be largely functionally redundant, CgYps1 has been demonstrated to be the sole CgYapsin that is required to survive low pH and menadione-induced oxidative stress (23, 27). Furthermore, a unique role of CgYps1 in acid and menadione stress survival has been associated with its regulation of the ATPase activity of the plasma membrane proton pump, CgPma1, and NADH:quinone oxidoreductase activity of the flavodoxin-like protein, CgPst2, respectively (23, 27). CgYps1 has also recently been reported to be enriched in the plasma membrane fraction of cell lysates, along with its substrate CgPst2 (27). Furthermore, we have recently reported an unexpected role of CgYapsins in secretome modulation as the secretome of *Cgyps1-11Δ* mutant (lacks all 11 CgYapsins) was found to be 4.6-fold larger, compared with the *wildtype* secretome (26). We also identified eight CgYapsins including CgYps1 in the *wildtype* secretome (26), underscoring the versatile and probably environmental cue-dependent location of CgYapsins.

In view of assorted physiological roles, and essentiality of CgYapsins for pathogenesis of *C. glabrata*, we are interested in deciphering the underlying molecular basis. Toward this end, we here have studied the effect of *CgYPS-11* gene loss on the composition of the total membrane proteome of logarithmic-phase *C. glabrata* cells and identified 262 and 189 proteins, *via* label-free quantitative membrane proteome analysis, which showed decreased and increased abundance in *Cgyps1-11Δ* mutant, respectively, as compared with *wildtype* cells. Furthermore, we correlated the high abundance of glycolysis proteins with impaired signaling of CgSnf3 (high-affinity glucose sensor)-dependent pathway, increased glucose uptake, and elevated fermentative metabolism in the *Cgyps1-11Δ* mutant. Altogether, our data unveil an unanticipated role of fungal GPI-anchored aspartyl proteases in regulating the extracellular glucose sensing mechanisms.

## Results

### Identification of global membrane proteomes of wildtype and Cgyps1-11Δ strains

We have recently shown that CgYapsins modulate the *C. glabrata* secretome, as the number of proteins secreted by the *Cgyps1-11Δ* mutant (lacks 11 putative GPI-linked aspartyl proteases) was 4.6-fold higher, compared with the *wildtype* (*wt*) strain (26). Since the conventional trafficking route, which proteins destined to be secreted undertake in membrane-bound vesicles, is from the endoplasmic reticulum to Golgi apparatus to cell membrane to the extracellular environment (28), we sought to extend our work further and profiled the total membrane proteome of the *Cgyps1-11Δ* mutant. For this, we performed global proteome analyses on total membrane fractions (TMFs) of log-phase grown cells of *wt* and *Cgyps1-11Δ* strains in duplicates. For global membrane proteomes, proteins that were identified in both biological replicate samples and represented by a minimum of two total peptides in each sample were selected for further analysis.

**Figure 1:**
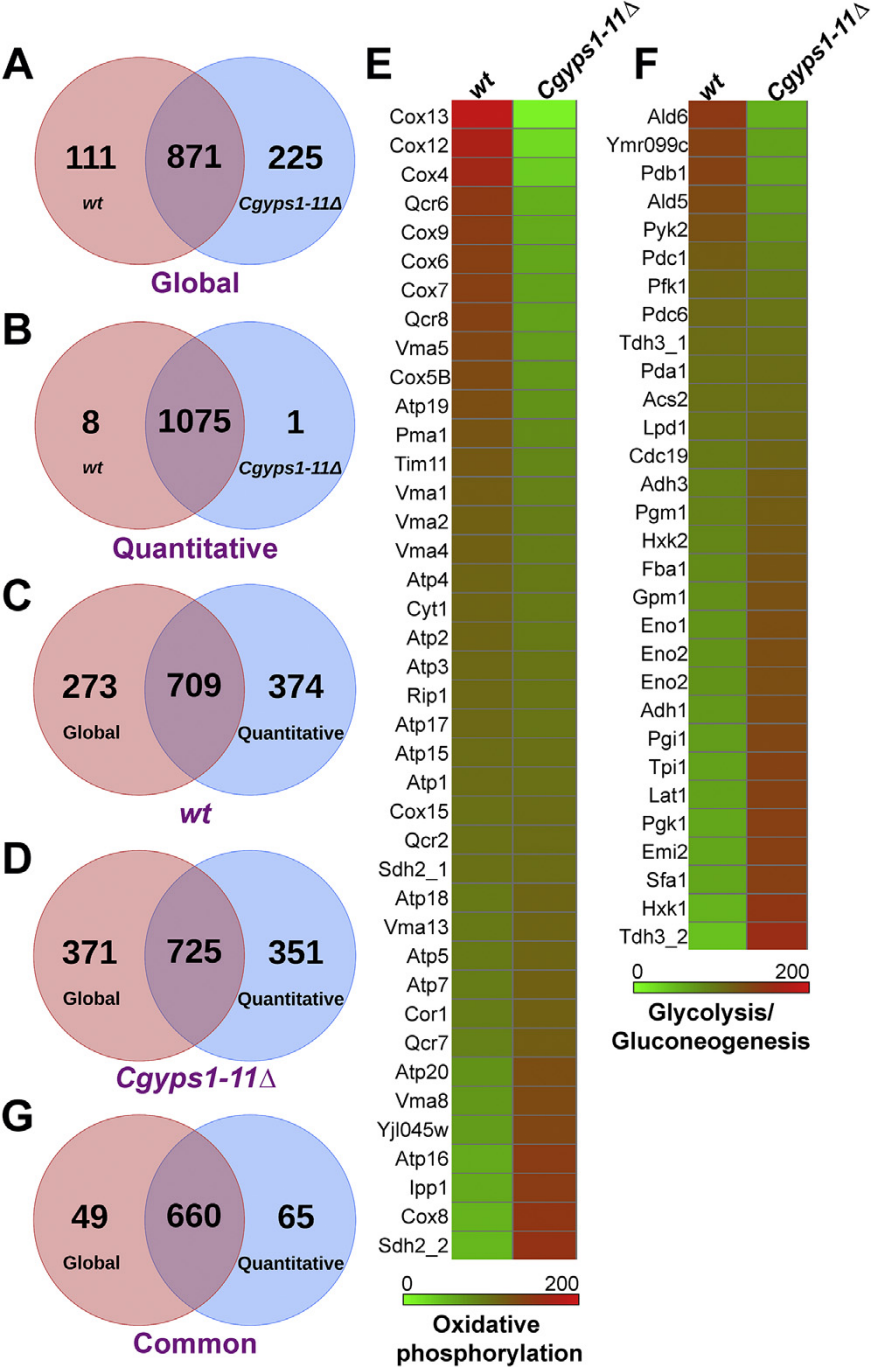
**Global membrane proteome analysis of *C. glabrata wildtype* and *Cgyps1-11Δ* strains.***A*, Venn diagram illustrating overlap between proteins identified in global membrane proteomes of *wildtype* (*wt*) and *Cgyps1-11Δ* strains. *B*, Venn diagram illustrating overlap between proteins identified in quantitative membrane proteomes of *wt* and *Cgyps1-11Δ* strains. *C*, Venn diagram illustrating overlap between proteins identified in global and quantitative membrane proteomes of the *wt* strain. *D*, Venn diagram illustrating overlap between proteins identified in global and quantitative membrane proteomes of the *Cgyps1-11Δ* mutant. *E* and *F*, heat map depicting abundance of proteins identified by quantitative membrane proteome profiling that belong to oxidative phosphorylation (*E*) and glycolysis/gluconeogenesis (*F*). Please note that *CAGL0C03223g* and *CAGL0E03850g* are labeled as Sdh2_1 and Sdh2_2, respectively, in *E*, and *CAGL0G09383g* and *CAGL0J00451g* are labeled as Tdh3_1 and Tdh3_2, respectively, in *F*. *G*, Venn diagram illustrating overlap between proteins identified in *wt* and *Cgyps1-11Δ* strains by both global and quantitative membrane proteome profiling.

A total of 982 and 1096 proteins were found to constitute the membrane proteomes of *wt* and *Cgyps1-11Δ* strains, respectively, with 871 proteins being common to both proteomes (Fig. 1*A* and Tables S1 and S2). Although Gene Ontology (GO) Slim analysis revealed that, of 5294 *C. glabrata* ORFs, the products of 1172 genes (20.9% of total genes) belong to the Cellular Component term “Membrane (GO:0016020)” (http://www.candidagenome.org/download/go/go_slim/C_glabrata_CBS138_go_distribution.tab), 1202 proteins, using computational, high-throughput, and manually curated methods, were found to be annotated as membrane proteins (http://www.candidagenome.org/cgi-bin/search/featureSearch). This difference in the protein number could be due to the kind of prediction/annotation tools used in the two analyses. Of 1202 annotated membrane proteins, 300 (30.55%) and 385 (35.13%) proteins were identified in global membrane proteomes of *wt* and *Cgyps1-11Δ* strains, respectively (Table S3). GO enrichment analysis of identified proteins in *wt* and *Cgyps1-11Δ* membrane proteomes using the FungiFun tool (https://elbe.hki-jena.de/fungifun/) revealed the identified proteins to belong to varied biological processes, including Translation, Intracellular protein transport, Oxidation-reduction process, Ergosterol biosynthetic process, Protein folding, Protein N-linked glycosylation, and Glycolytic process (Tables S4 and S5). The subcellular localization of all identified proteins was predicted using the DeepLoc 1.0 Eukaryotic protein subcellular localization predictor tool (http://www.cbs.dtu.dk/services/DeepLoc-1.0/index.php), and the primary localization of 982 and 1096 proteins identified in *wt* and *Cgyps1-11Δ* membrane proteomes, respectively, is summarized in Table S6.

The membrane proteome of a *C. glabrata* strain, ATCC 66032, has previously been identified, using the iTRAQ labeling approach, wherein a total of 624 proteins were identified in minimal medium–grown cultures (29). Of these 624 proteins, 361 (57.85%) and 407 (65.22%) proteins were present in membrane proteomes of *wt* and *Cgyps1-11Δ* strains, respectively, profiled in our study (Table S7), indicating a good overlap between the published and our dataset. The differences in proteins recovered in both analyses could be due to the usage of two different strains and rich/minimal growth medium, and sample preparation and analysis methods.

### Comparative analysis of wt and Cgyps1-11Δ membrane proteomes

The strain-wise comparison of proteins identified in total membrane fractions of the *wt* and *Cgyps1-11Δ* strains revealed 871 common proteins, indicating 80% overlap between the two strains, with 111 and 225 proteins being exclusively present in the membrane proteomes of *wt* and *Cgyps1-11Δ* mutant, respectively (Fig. 1*A* and Tables S1 and S2). The enrichment analysis of GO terms in strain-specific proteins, using the DAVID Functional Annotation Tool (https://david.ncifcrf.gov/tools.jsp), showed 42-fold enrichment of GO:0033215 (iron assimilation by reduction and transport) and 12-fold enrichment of GO:0006491 (N-glycan processing) terms, in *wt* and *Cgyps1-11Δ* membrane proteomes, respectively (Table S8). This exclusive presence of 111 and 225 proteins in *wt* and *Cgyps1-11Δ* membrane proteomes, respectively, could arise from drastic changes in the protein abundance in the absence of 11 proteases. Therefore, we next conducted the label-free quantitative membrane proteomics to identify proteins whose levels were altered in the *Cgyps1-11Δ* mutant.

### Label-free quantitative proteome analysis of membrane fractions of wt and Cgyps1-11Δ strains

Using label-free quantitative proteomics, we identified proteins in total membrane fractions, prepared from lysates of the log-phase grown *wt* and *Cgyps1-11Δ* cells, that showed differential abundance (≥1.5-fold change in levels) in the *Cgyps1-11Δ* mutant. This label-free membrane protein quantification analysis yielded 1083 and 1076 proteins that were identified in *wt* and *Cgyps1-11Δ* strains, respectively. A set of 1075 proteins was common between *wt* and *Cgyps1-11Δ* membrane fractions, whereas 8 and 1 proteins were present uniquely in *wt* and *Cgyps1-11Δ*, respectively (Fig. 1*B* and Table S9). Among 1075 proteins identified in TMFs of both *wt* and *Cgyps1-11Δ* strains, 1004 and 71 proteins were identified with high (<0.01 FDR) and medium (<0.05 FDR) confidence, respectively. The set of nine proteins, identified uniquely in the *wt* or *Cgyps1-11Δ* strain, was identified with high confidence. Of note, 22.2% (241) and 5% (54) of all identified proteins (1084) belonged to the *Candida* GO-SLIM Cellular Component term “Membrane” and “Plasma membrane,” respectively (http://www.candidagenome.org/cgi-bin/GO/goTermMapper). Similar to the global membrane proteome analysis, GO enrichment analysis of 1084 proteins identified in the label-free quantitative membrane proteomics, using the FungiFun tool, revealed the proteins to be involved in various processes including Translation, Glycolytic process, Oxidation-reduction process, rRNA processing, Tricarboxylic acid cycle, Intracellular protein transport, and Protein folding (Table S10).

**Supplemental Figure S1:**
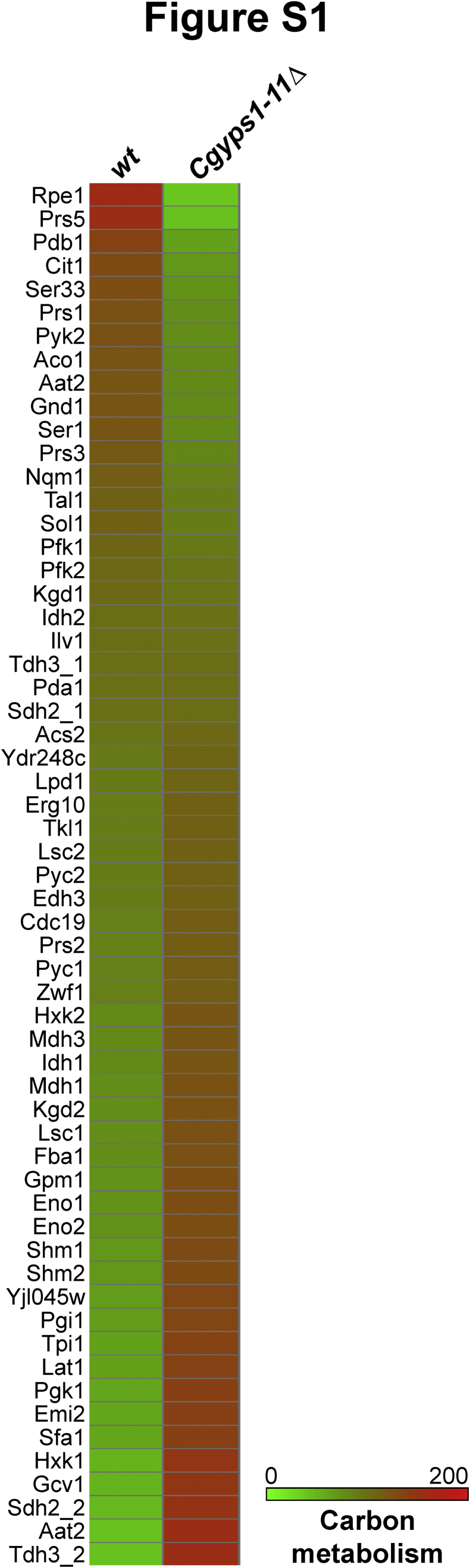


Furthermore, of 1075 proteins, common to both *wt* and *Cgyps1-11Δ* strains, we identified proteins whose levels were altered by ≥1.5-fold in the *Cgyps1-11Δ* mutant. We found 262 and 189 proteins to display decreased and increased abundance, respectively, in TMF of the *Cgyps1-11Δ* mutant as compared with the *wt* TMF (Tables S11 and S12), whereas 624 proteins showed no change in abundance (Table S13). Therefore, overall, *CgYPS-11* gene loss altered the abundance of 42% of identified total membrane proteins. GO analysis of differentially abundant membrane proteins, using the FungiFun and DAVID tools, revealed the enrichment of Intracellular protein transport, Ergosterol biosynthetic process and Mitochondrial electron transport, and Glycolytic process and Gluconeogenesis processes in proteins with lower and higher abundance, respectively, in the *Cgyps1-11Δ* mutant (Tables S14 and S15). To probe deeper into glucose metabolism proteins, we next performed the KEGG pathway analysis on all 1084 identified proteins and found 59 (5.4%), 40 (3.7%), and 30 (2.8%) proteins to belong to Carbon metabolism, Oxidative phosphorylation, and Glycolysis/Gluconeogenesis processes, respectively, and the abundance of these proteins in *wt* and *Cgyps1-11Δ* cells is shown as heat maps in Fig. S1 and Figure 1, *E* and *F*, respectively.

Next, we compared the global and quantitative membrane proteomes and found a set of 709 (∼72%) and 725 (∼66%) proteins to be common between the global and quantitative membrane proteomes of *wt* and *Cgyps1-11Δ* strains, respectively (Fig. 1, *C* and *D*). Among these 709 and 725 proteins, 660 proteins were common between both strains (Fig. 1*G* and Tables S16–S18). Of note, this set of 660 proteins detected in both global and quantitative membrane proteomes of *wt* and *Cgyps1-11Δ* strains are likely to represent *bona fide* membrane proteins that are expressed in YPD medium–grown log-phase *C. glabrata* cells. Moreover, the CGD GO Slim Mapper tool predicted 24% (161) of these 660 proteins to be membrane proteins, with 18.5%, 15.9%, and 15.5% proteins being associated with hydrolase activity, RNA binding, and transferase activity, respectively. Of importance, 25.8% (170) of these 660 proteins were mapped to the GO Biological Process Slim term “Transport,” indicating that a quarter of the identified membrane proteins are likely to be involved in the movement of molecules across membranes.

### Mitochondrial morphology is not perturbed in the Cgyps1-11Δ mutant

**Figure 2:**
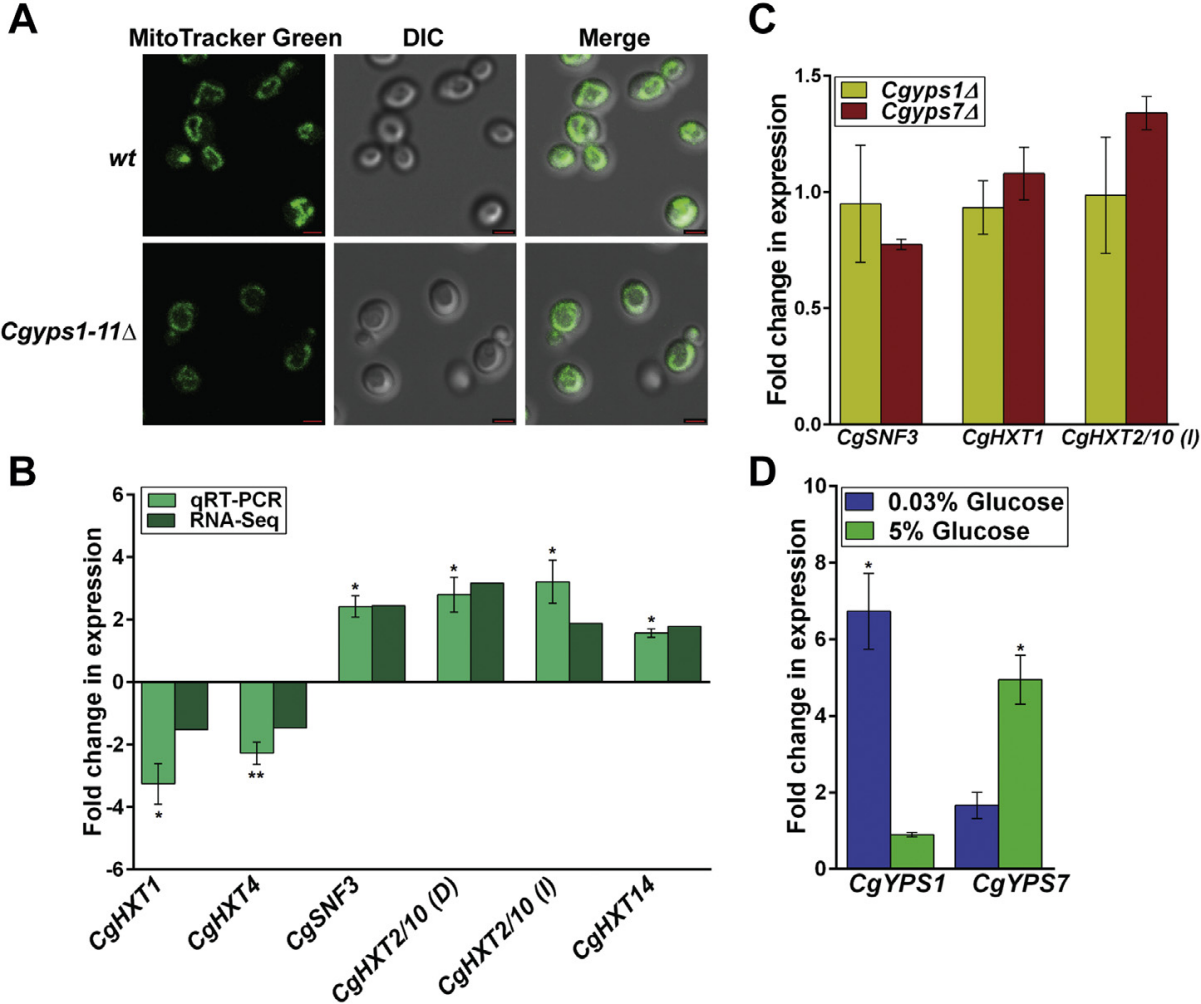
**Glucose sensing and transport genes are deregulated in the *Cgyps1-11Δ* mutant.***A*, MitoTracker Green–based mitochondrial morphology analysis. Representative maximum-intensity projection of Z-stack fluorescence confocal images showing mitochondrial network in YNB medium–grown, MitoTracker Green (100 nM)–stained log-phase cells of *wt* and *Cgyps1-11Δ* strains. The scale bar represents 2 μm. *B*, qRT-PCR-based expression analysis of indicated genes (two downregulated and four upregulated genes in the RNA-Seq experiment) in *wt* and *Cgyps1-11Δ* strains. Strains were grown to log-phase in YPD medium for 4 h, followed by RNA extraction using acid phenol. The cDNA synthesis reaction was set up with 500 ng total RNA, followed by qRT-PCR using appropriate primer sets, and gene transcript levels were measured using the 2^−ΔΔCt^ method. Please note that *CgHXT2/10* (*D*) and *CgHXT2/10* (*I*) refer to *CAGL0D02640g/CAGL0D02662g* and *CAGL0I00286g* genes, respectively. Data (mean ± SEM, n = 3–4) were normalized against *CgACT1* mRNA control and represent fold change in expression in *Cgyps1-11Δ* cells, compared with *wt* cultures (considered as 1.0). ∗*p* ≤ 0.05; ∗∗*p* ≤ 0.01, paired two-tailed Student's *t* test. *C*, qRT-PCR-based expression analysis of indicated genes in YPD medium–grown log-phase *Cgyps1Δ* and *Cgyps7Δ* cells. Data (mean ± SD, n = 2–3) were normalized against *CgACT1* mRNA control and represent fold change in expression in *Cgyps1Δ* and *Cgyps7Δ* cells, compared with *wt* cultures (considered as 1.0). *D*, qRT-PCR-based expression analysis of *CgYPS1* and *CgYPS7* genes in log-phase *wt* cells that were grown in YNB medium containing low (0.03%), regular (2%), and high (5%) glucose for 2 h. Data (mean ± SEM, n = 3–4) were normalized against *CgACT1* mRNA control and represent fold change in expression in low-glucose and high-glucose medium–grown *wt* cells, compared with regular-glucose medium–grown *wt* cells (considered as 1.0). ∗*p* ≤ 0.05, paired two-tailed Student's *t* test. DIC, differential interference contrast.

From the GO biological process enrichment analysis, we first focused on the mitochondrial electron transport (ETP) and pulled out proteins that are implicated in this process and were underrepresented in the membrane proteome of the *Cgyps1-11Δ* mutant (Fig. 1*E* and Table S11). These ETP proteins also included seven CgCox proteins, CgCox4, CgCox5B, CgCox6, CgCox7, CgCox9, CgCox12, and CgCox13 (Table S11), which represented different subunits of the cytochrome C oxidase (COX). Of note, COX (complex IV) is the terminal oxidase of the ETP chain in the inner membrane of the mitochondria, which reduces oxygen to produce water and facilitates ATP production (30). In this context, it is noteworthy that the *Saccharomyces cerevisiae* mutant lacking *COX4* gene is known to have diminished viability in stationary phase and altered mitochondrial reticular network (31). Since the *Cgyps1-11Δ* mutant has previously been shown to lose viability in stationary phase growth conditions (5), we decided to examine the mitochondrial morphology, with the rationale that the low abundance of Cox proteins in *Cgyps1-11Δ* mutant may lead to a perturbation in the mitochondrial architecture. For this, we stained *Cgyps1-11Δ* and *wt* cells with the MitoTracker Green dye, as its localization to the mitochondria is independent of the mitochondrial membrane potential. We found robust mitochondrial network in both strains (Fig. 2*A*), which ruled out the possibility of any gross morphology defect in the mitochondrial structure upon loss of CgYps1–11 aspartyl proteases. Of note, since the *Cgyps1-11Δ* mutant is able to utilize nonfermentable carbon sources, glycerol and ethanol (23), the significance of diminished levels of Cox proteins on mitochondrial physiology is yet to be determined.

### Genes involved in glucose sensing and transport are deregulated in the Cgyps1-11Δ mutant

Next, we focused on glycolytic process, identified in the GO biological process enrichment analysis, and pulled out proteins involved in this process that were overrepresented in the membrane proteome of the *Cgyps1-11Δ* mutant, as compared with the *wt* membrane proteome (Fig. 1*F* and Table S12). These proteins included enzymes, CgGpm1, CgPgi1, CgTpi1, CgFba1, CgPgk1, CgEno1, and CgEno2, that carry out different enzymatic reactions during breakdown of glucose by glycolysis, with some also performing the reverse reactions during glucose synthesis (22, 32). The enrichment of glycolysis and gluconeogenesis proteins in the mutant membrane proteome was particularly intriguing, in light of the decreased abundance of three hexose transporters, CgHxt1, CgHxt4, and CgHxt5, in the membrane proteome (Tables S11 and S19), and reported transcriptome profiles of the *Cgyps1-11Δ* mutant (19). We have previously analyzed the transcriptomes of log-phase cultures of *wt* and *Cgyps1-11Δ* strains by RNA-Seq approach and found genes involved in ion transport and sterol import to be downregulated and genes involved in carbohydrate metabolism, cell wall organization, and tricarboxylic acid cycle to be upregulated in the mutant (19).

Therefore, we next compared the quantitative membrane proteome results with RNA-Seq transcriptome data, wherein we found differential expression of three genes, *CgSNF3* (*CAGL0J09020g*), *CgHXT1* (*CAGL0A01804g*), and *CgHXT14* (*CAGL0M04103g*), that code for the high-affinity glucose sensor, hexose transporter, and hexose transporter, respectively, in the *Cgyps1-11Δ* mutant (19). These genes have previously been implicated in glucose sensing in *C. glabrata* (33, 34). Of the three genes, *CgSNF3* and *CgHXT14* were upregulated, whereas *CgHXT1* was downregulated (19). Since CgHxt1 also displayed decreased abundance in the total membrane proteome of the *Cgyps1-11Δ* mutant (Table S11), these data suggest that both gene and protein levels of *CgHXT1* are downregulated in the mutant.

Ng *et al.* (33) have previously identified the sugar-receptor repressor pathway genes in *C. glabrata*, which are likely to be involved in glucose sensing. These include 11 hexose transporters, two glucose sensors CgSnf3 and CgRgt2, and two transcriptional regulators CgRgt1 and CgMig1 ((33, 34, 35); Table S19). For validation of our published RNA-Seq data by quantitative RT-PCR (qRT-PCR), we selected the aforementioned three genes, whose expression was differentially regulated in the RNA-Seq-based transcriptome profiling of the *Cgyps1-11Δ* mutant cells (19), as well as three other hexose transporter genes, *CgHXT4* (*CAGL0A01782g*), *CgHXT2/10* (*D*) (*CAGL0D02640g/CAGL0D02662g*), and *CgHXT2/10* (*I*) (*CAGL0I00286g*), with *CAGL0D02640g* and *CAGL0D02662g* genes sharing 100% nucleotide identity ((33, 35); http://www.candidagenome.org/). The rationale behind selecting the latter set of three genes was their differential transcript levels in the *Cgyps1-11Δ* mutant; however, these were not classified as differentially expressed genes in our RNA-Seq analysis, as these did not meet one of the applied cutoff criteria, *viz.*, ≥1.5-fold change in expression and a false discovery rate–adjusted *p* value of ≤0.05 to the RNA-Seq data (19). We found 2.8- and 2.3-fold lower transcript levels of *CgHXT1* and *CgHXT4* genes, respectively, whereas the expression of *CgSNF3*, *CgHXT2/10* (*D*) (*CAGL0D02640g/CAGL0D02662g*), *CgHXT2/10* (*I*) (*CAGL0I00286g*), and *CgHXT14* genes was increased by 1.6- to 3.2-fold in the *Cgyps1-11Δ* mutant, compared with the *wt* strain (Fig. 2*B*). These data suggest that the loss of CgYapsins results in deregulated expression of genes involved in glucose sensing and uptake.

Since, of the 11 CgYapsins, CgYps1 and CgYps7 predominantly regulate many processes *in vitro* (19, 23, 24), we next determined whether the single deletion of *CgYPS1* or *CgYPS7* gene could lead to the perturbed expression of *CgSNF3* and hexose transporter genes, *CgHXT1* (*CAGL0A01804g*) and *CgHXT2/10* (*I*) (*CAGL0I00286g*). We found transcript levels of *CgSNF3*, *CgHXT1*, and *CgHXT2/10* (*I*) genes to be similar in *wt* and *Cgyps1Δ* and *Cgyps7Δ* mutants (Fig. 2*C*), indicating that *CgYPS1* or *CgYPS7* gene loss singly has no effect on the transcriptional regulation of these glucose homeostasis genes. This result could in part be due to functional redundancy among CgYapsins, as reported previously (19, 24).

Furthermore, to investigate the role of CgYapsins in cellular response to extracellular glucose, we checked if *CgYPS1* and *CgYPS7* gene expression is regulated by glucose concentration in the medium. Of note, *CgYPS1* transcription is known to be activated in response to low pH and high temperature (23, 36). Since the rich YPD and synthetically defined minimal YNB media, used for routine culturing of *C. glabrata* strains, contain 2% glucose as carbon source, we used 0.03% and 5% glucose as glucose-limited and glucose-excess growth conditions, respectively. In this regard, it is noteworthy that 0.01% and 2% glucose have previously been reported as low-glucose and high-glucose environment, respectively, for *C. glabrata* (33). Transcript measurement by qRT-PCR revealed 7-fold activation of *CgYPS1* gene upon exposure to low-glucose medium, and 5-fold activation of *CgYPS7* gene in high-glucose medium (Fig. 2*D*). These data suggest that the cellular transcriptional response to environmental glucose levels involves activation of *CgYPS1* and *CgYPS7* genes, with *CgYPS1* and *CgYPS7* being activated during glucose-limited and glucose-excess conditions, respectively. In this context, it is noteworthy that *C. glabrata* is a crab-tree positive yeast, which prefers the fermentation mode of growth even in the presence of oxygen (22), and is assumed to encounter a glucose-limited environment in host phagocytic cells, including macrophages and neutrophils, with CgYps1 playing a key role in survival of the macrophage internal milieu (5, 17, 19, 37). Altogether, our data suggest that *C. glabrata* cells respond to glucose-limited and surplus conditions by activating the expression of *CgYPS1* and *CgYPS7* genes, respectively, and the *Cgyps1-11Δ* mutant, lacking 11 proteases, displays deregulated expression of genes involved in glucose sensing and transport.

### CgSNF3 is required for virulence of C. glabrata

CgSnf3 is a high-affinity glucose sensor that is involved in sensing and transducing the low extracellular glucose concentration, and its loss is known to result in the transcriptional repression of four hexose transporter genes (34). *CgSNF3* itself is transcriptionally activated in response to low extracellular glucose concentration (33). Therefore, an increased expression of the *CgSNF3* gene in the YPD medium (contains 2% glucose)–grown *Cgyps1-11Δ* cells prompted us to examine whether CgSnf3-mediated signaling of glucose-limited environment is impaired in the mutant. We reasoned that, if elevated expression of genes coding for glucose transporters in the *Cgyps1-11Δ* mutant is due to higher *CgSNF3* transcript levels, deletion of the *CgSNF3* gene in the mutant strain could lead to decreased expression of *HXT* genes. For this, we created two deletion strains, *Cgsnf3Δ* (*CgSNF3*-deleted in *wt* background) and *Cgsnf3Δyps1-11Δ* (deleted for 12 genes, *CgSNF3* and *CgYPS1–11*), and first characterized the *Cgsnf3Δ* mutant.

**Figure 3:**
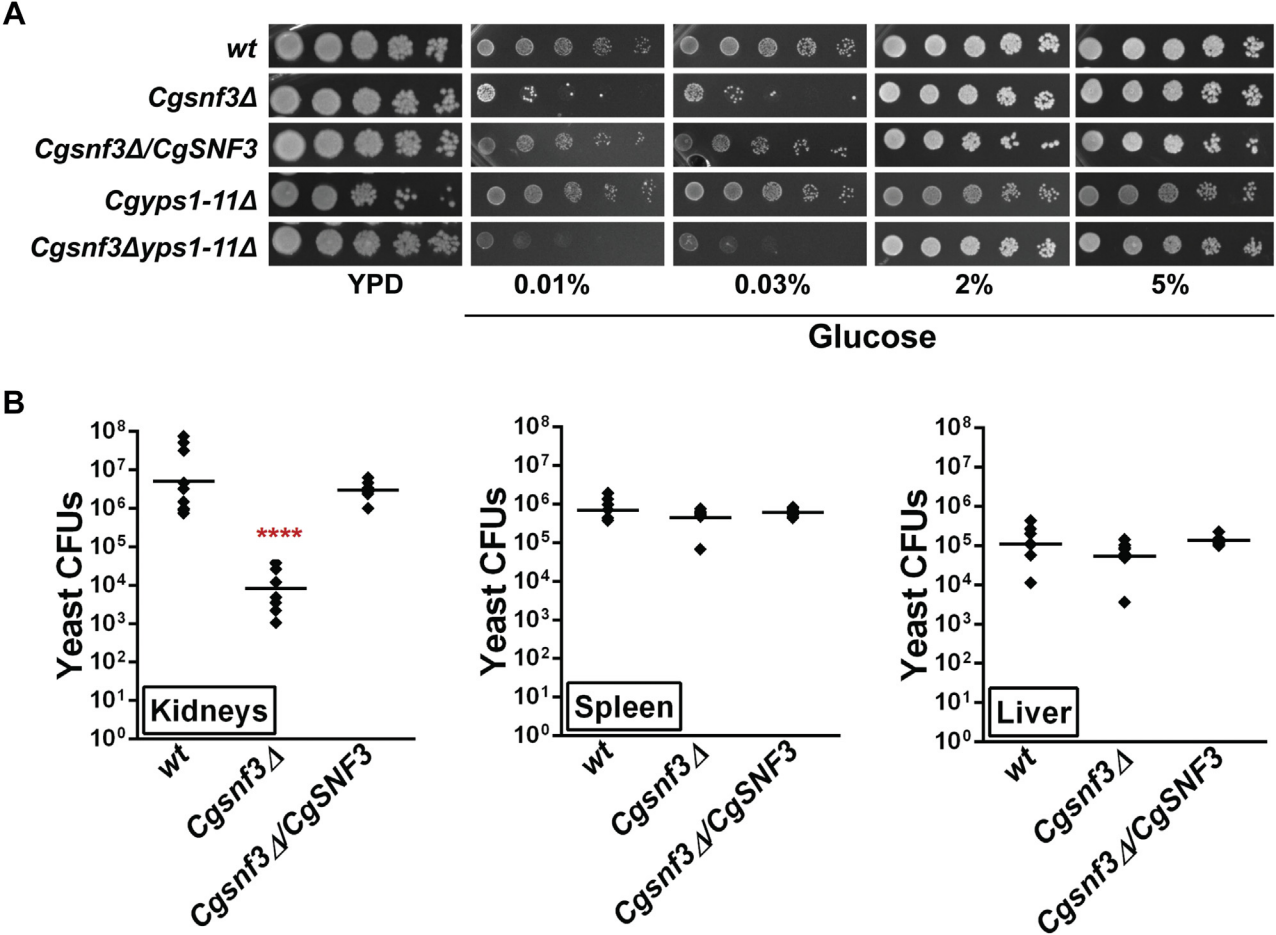
***CgSNF3* is required for virulence.***A*, serial dilution spotting analysis of *wt*, *Cgsnf3Δ*, *Cgsnf3Δ/CgSNF3*, *Cgyps1-11Δ*, and *Cgsnf3Δyps1-11Δ* strains in YPD (2% dextrose) or YNB medium containing indicated glucose concentrations. *C. glabrata* cultures were grown overnight in casamino acid medium and normalized to *A*_600_ of 1.0. After diluting cultures 10-fold serially in PBS, 3 μl was spotted on YPD medium or YNB medium containing 0.01%, 0.03%, 2%, and 5% glucose. Plates were incubated at 30 °C, and images were captured after 2 days. *B*, colony-forming unit–based survival analysis of the *Cgsnf3Δ* mutant. *C. glabrata* strains (100 μl cell suspension; 4 × 10^7^ cells) were infected into the tail vein of 6- to 8-week-old female BALB/c mice. After 7 days, mice were sacrificed and three organs (kidneys, liver, spleen) were collected and homogenized in PBS. The homogenates were diluted in PBS, and appropriate dilutions were plated on penicillin- and streptomycin-containing YPD medium. The colony-forming units (CFUs) recovered from each organ of the individual mouse are represented by diamonds in graphs. The *horizontal line bars* represent the CFU geometric mean (n = 7–9) for each organ. Statistically significant differences in CFUs between kidneys of *wt*- and *Cgsnf3Δ*-infected mice are marked. ∗∗∗∗*p* < 0.0001; Mann-Whitney U test.

CgSnf3 has previously been shown to be required for growth in low-glucose medium (34); we, therefore, first verified phenotypes of the generated *Cgsnf3Δ* mutant strain. Similar to the earlier study (34), we found growth of the *Cgsnf3Δ* mutant to be highly attenuated in glucose-limited medium (0.01% and 0.03% glucose), which was restored upon ectopic expression of the *CgSNF3* gene (Fig. 3*A*).

The *Cgsnf3Δ* mutant has previously been shown to form robust biofilms but diminished survival in macrophages (34). Therefore, to delineate the role of CgSnf3 *in vivo*, we checked the survival of the *Cgsnf3Δ* mutant in a murine model of systemic candidiasis. We recovered about 1000-fold lower fungal colony-forming units from kidneys of *Cgsnf3Δ*-infected BALB/c mice, compared with *wt*-infected BALB/c mice (Fig. 3*B*). This reduced renal fungal load was alleviated in mice infected with the *Cgsnf3Δ* mutant expressing *CgSNF3* ectopically (Fig. 3*B*), indicating that the diminished fungal survival in murine kidneys is due to the lack of the *CgSNF3* gene. Of note, since the glucose abundance is known to modulate the expression of various fungal pathogenesis-associated factors, including morphogenesis, biofilm formation, and stress resistance genes (22, 38, 39, 40), it is possible that both deregulated expression of such factors and impaired glucose homeostasis contribute to the diminished survival of the *Cgsnf3Δ* mutant in murine kidneys. Important, the fungal burden in liver and spleen was the same in both *wt* and *Cgsnf3Δ* infected mice (Fig. 3*B*). Therefore, besides verifying the role of CgSnf3 in growth in glucose-limited conditions, our data implicate for the first time CgSnf3 in virulence in mice in an organ-dependent manner.

### CgSNF3 deletion rescues the growth defect of the Cgyps1-11Δ mutant in 2% glucose–containing medium

**Supplemental Figure S2:**
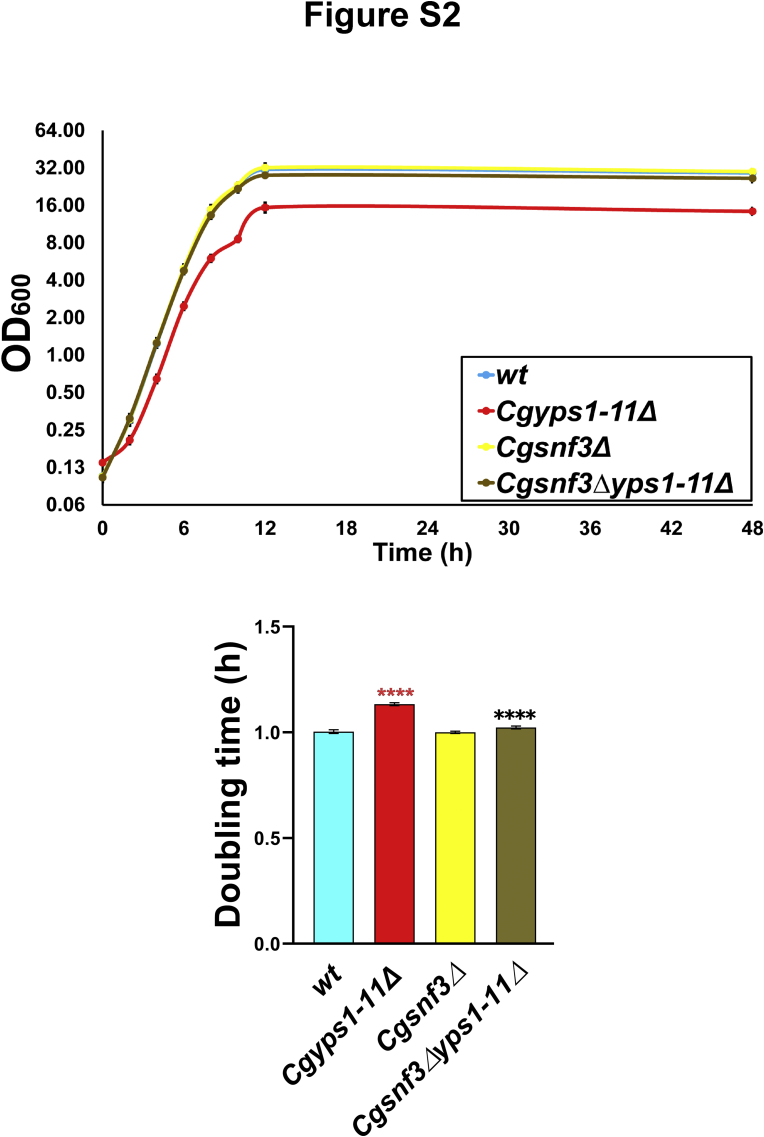


Next, we characterized the *Cgsnf3Δyps1-11Δ* mutant lacking 11 CgYapsins and CgSnf3 glucose sensor. We found this mutant to be growth defective in low-glucose medium, similar to the *Cgsnf3Δ* mutant (Fig. 3*A*). During phenotypic characterization of the generated mutants, we noticed that the *Cgsnf3Δyps1-11Δ* mutant grew better in 2% dextrose-containing YPD and YNB media, than the *Cgyps1-11Δ* mutant (Figs. 3*A* and S2), which is known to be a slow grower (5), indicating that *CgSNF3* deletion confers a growth advantage to the *Cgyps1-11Δ* mutant. One possible reason for this better growth could be high basal transcript levels of *CgSNF3*, and the consequent activation of the high-affinity glucose uptake pathway even in the presence of surplus glucose in the environment. Consistent with this notion, growth attenuation of the *Cgyps1-11Δ* mutant, compared with the *wt* strain, was much less in medium containing low glucose (Fig. 3*A*). These data suggested that the slow growth phenotype of the *Cgyps1-11Δ* mutant is specific to the high-glucose medium and could be due to deregulated CgSnf3-mediated glucose signaling.

**Figure 4:**
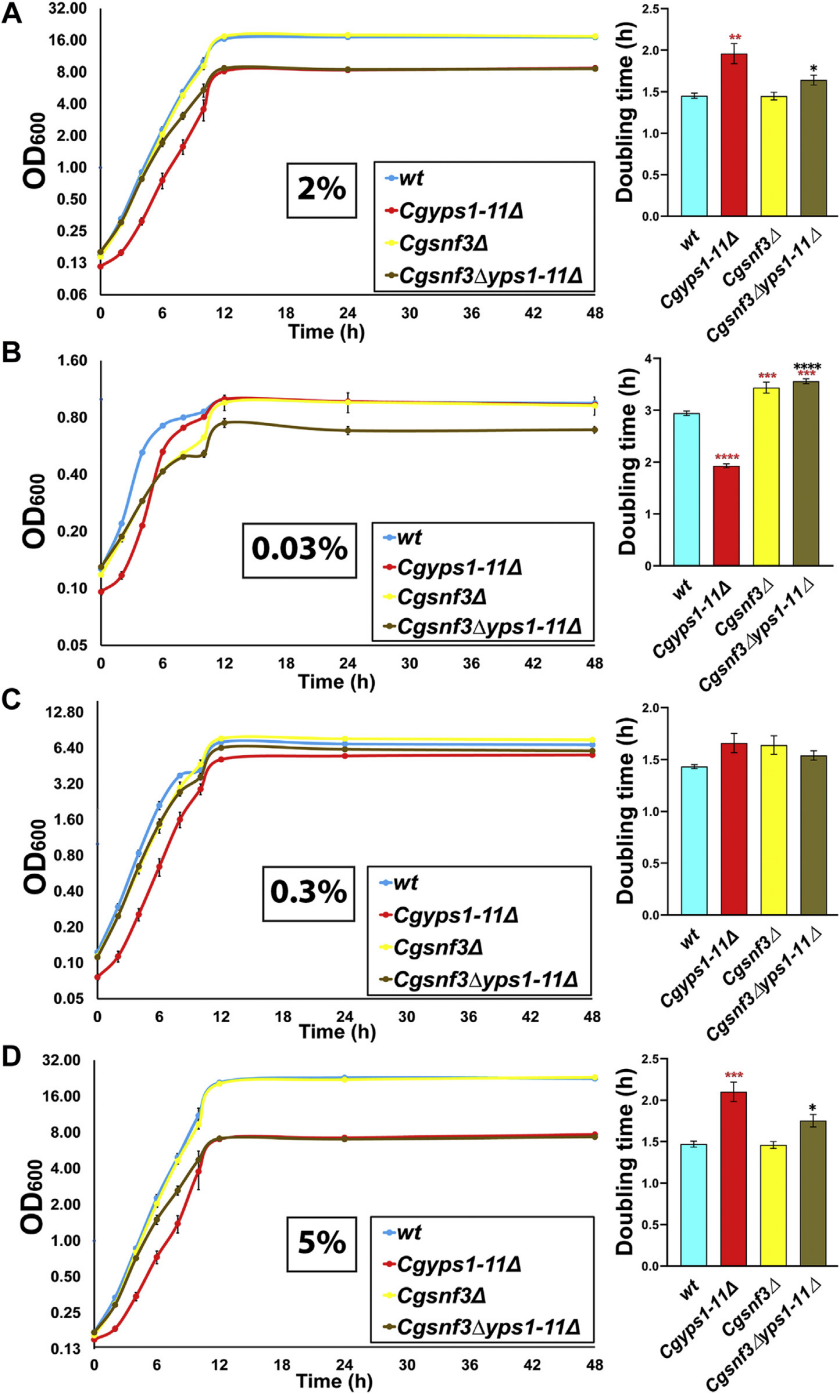
***CgSNF3* gene loss confers growth advantage to *Cgyps1-11Δ* cells in medium containing 2% glucose.** Time course analysis of *wt*, *Cgyps1-11Δ*, *Cgsnf3Δ*, and *Cgsnf3Δyps1-11Δ* strains. *C. glabrata* strains were grown overnight in YPD medium and inoculated at an initial *A*_600_ of 0.1 in YNB medium containing 2% (*A*), 0.03% (*B*), 0.3% (*C*), and 5% (*D*) glucose. Cultures were incubated at 30 °C with continuous shaking (200 rpm), and absorbance was monitored at regular intervals till 48 h. The absorbance (*A*_600_) values are plotted against time, and the growth period, corresponding to the log-phase (between 2 and 6 h), was used to determine the doubling time. Data represent mean ± SEM (n = 3–4). The one-way ANOVA with Tukey’s test was employed to determine the statistical significance of doubling time differences between strains. *Red* and *black asterisks* denote differences in doubling time between *wt* and mutants, and *Cgyps1-11Δ* and *Cgsnf3Δyps1-11Δ* mutants, respectively. ∗*p* ≤ 0.05; ∗∗*p* ≤ 0.01; ∗∗∗*p* ≤ 0.001; ∗∗∗∗*p* ≤ 0.0001.

Therefore, to test this hypothesis, we next verified mutants’ growth profiles by conducting liquid medium–based growth time-course analyses in YNB medium containing 0.03%, 0.3%, 2%, and 5% glucose over the 48-h period. Expectedly, we found that, compared with *wt* cells, the *Cgyps1-11Δ* mutant exhibited 1.3-fold higher doubling time in YNB medium containing 2% glucose (Fig. 4*A*), whereas the *Cgsnf3Δ* mutant displayed 1.2-fold higher doubling time in YNB medium containing 0.03% glucose (Fig. 4*B*). In contrast, the growth rate of the *Cgyps1-11Δ* and *Cgsnf3Δ* mutants was same as that of the *wt* strain in glucose-limited (0.3%) and glucose-rich (5%) medium, respectively (Fig. 4, *C* and *D*). Intriguing, the doubling time of the *Cgsnf3Δyps1-11Δ* mutant population was substantially lower and higher than that of the *Cgyps1-11Δ* mutant population in medium containing 2% and 0.03% glucose, respectively (Fig. 4, *A* and *B*). Moreover, a pivotal requirement for CgSnf3 for growth in glucose-deficient medium was underscored by similar doubling times of the *Cgsnf3Δ* and *Cgsnf3Δyps1-11Δ* mutants in 0.03% glucose medium (Fig. 4*B*). Collectively, these data suggest that elevated environmental glucose levels lead to growth retardation in the *Cgyps1-11Δ* mutant and implicate CgYapsins in maintenance of glucose homeostasis probably *via* regulation of CgSnf3-dependent glucose sensing and signaling pathway.

### Transcriptional response to extracellular glucose is impaired in the Cgyps1-11Δ mutant

To probe deeper into CgSnf3-mediated glucose signaling in *C. glabrata*, we next examined the transcript levels of *CgSNF3*; three hexose transporter genes, *CgHXT1*, *CgHXT2/10* (*I*), and *CgHXT3* (*CAGL0A02321g*); *CgRGT1* (codes for a glucose-responsive transcription factor that is regulated by the low-affinity glucose sensor CgRgt2); and *CgMIG1* (encodes transcription factor involved in glucose repression) genes (Table S19) in all four mutant strains under low- (0.03%), regular- (2%), and high- (5%) glucose conditions. The rationale underlying the selection of these genes was their transcriptional regulation either in response to external glucose concentration or *CgSNF3/CgYPS1–11* deletion. Of note, *CgSNF3*, *CgRGT1*, and *CgHXT3* are known to be transcriptionally activated under low-glucose conditions while *CgHXT1* and *CgHXT3* are downregulated and upregulated, respectively, in the *Cgsnf3Δ* mutant (33, 34). Similarly, the *CgMIG1* gene has been reported to be expressed at higher levels in 2% dextrose medium, as compared with no-glucose medium (33). Last, as presented earlier (Fig. 2*B*), *CgSNF3* and *CgHXT2/10*, and *CgHXT1* genes are induced and repressed, respectively, in the *Cgyps1-11Δ* mutant.

**Figure 5:**
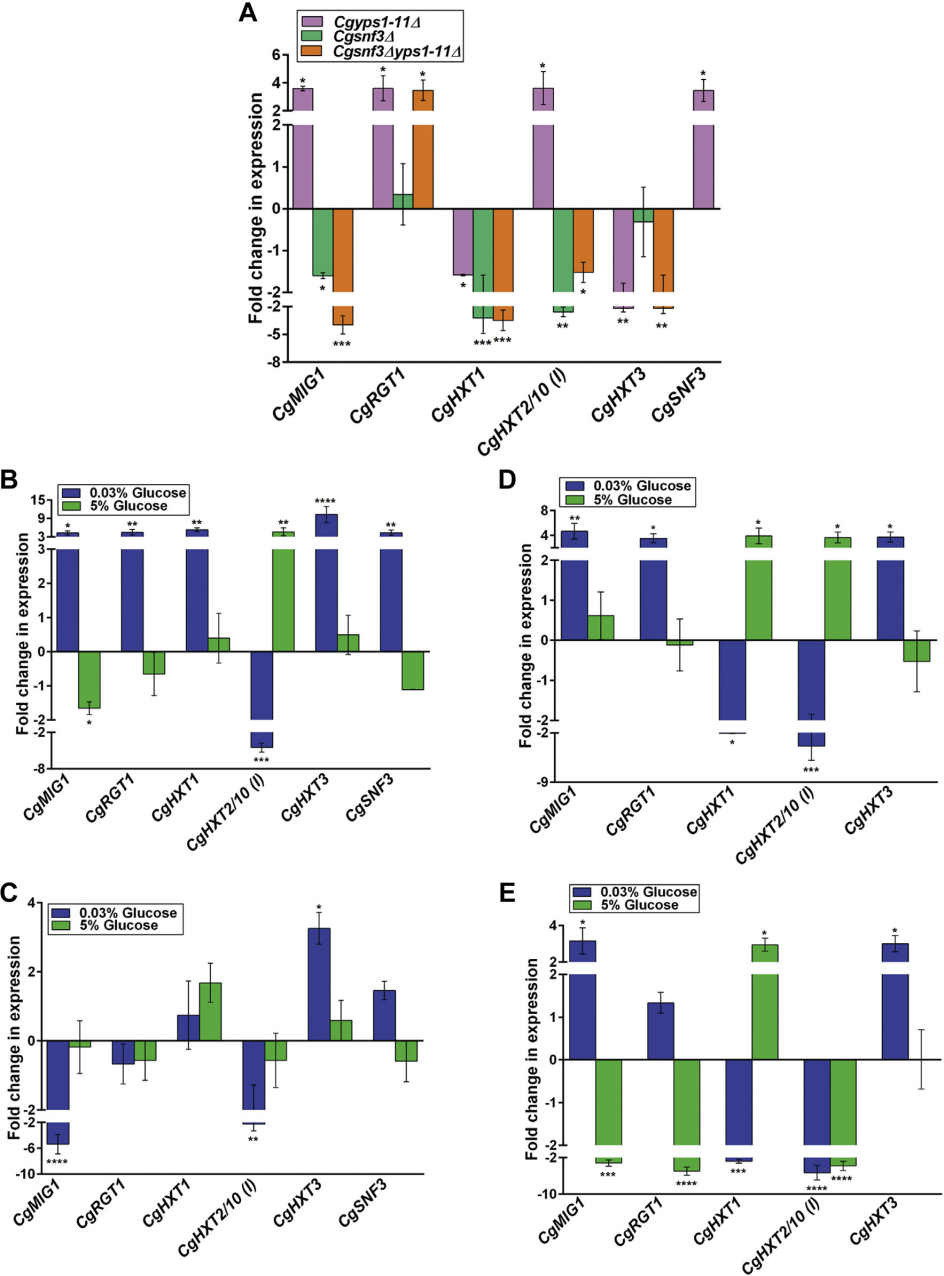
**The response to extracellular glucose is impaired in the *Cgyps1-11Δ* mutant.***A*, qRT-PCR-based expression analysis of indicated genes in YNB medium–grown log-phase *wt*, *Cgyps1-11Δ*, *Cgsnf3Δ*, and *Cgsnf3Δyps1-11Δ* cells. Data (mean ± SEM, n = 3–4) were normalized against *CgACT1* mRNA control and represent fold change in expression in mutant cells, compared with *wt* cultures (considered as 1.0). ∗*p* ≤ 0.05; ∗∗*p* ≤ 0.01; ∗∗∗*p* ≤ 0.001, one-way ANOVA with uncorrected Fisher’s LSD test. *B*–*E*, qRT-PCR-based expression analysis of indicated genes in log-phase *wt* (*B*), *Cgyps1-11Δ* (*C*), *Cgsnf3Δ* (*D*), and *Cgsnf3Δyps1-11Δ* (*E*) cells that were grown in YNB medium containing low (0.03%), regular (2%), and high (5%) glucose for 2 h. Data (mean ± SEM, n = 3–4) were normalized against *CgACT1* mRNA control and represent fold change in expression in low-glucose and high-glucose medium–grown cells, compared with regular-glucose medium–grown cells of each strain (considered as 1.0). ∗*p* ≤ 0.05; ∗∗*p* ≤ 0.01; ∗∗∗*p* ≤ 0.001; ∗∗∗∗*p* ≤ 0.0001, one-way ANOVA with uncorrected Fisher’s LSD test.

As shown in Figure 5*A*, we found *CgMIG1*, *CgRGT1*, *CgSNF3*, and *CgHXT2/10* (*I*) gene expression to be upregulated, whereas the transcription of *CgHXT1* and *CgHXT3* genes was downregulated in YNB medium–grown *Cgyps1-11Δ* cells, compared with YNB medium–grown *wt* cells (Fig. 5*A*). Contrarily, transcript levels of *CgMIG1* and *CgHXT2/10* genes were lower in the *Cgsnf3Δ* mutant, compared with *wt* cells (Fig. 5*A*). Furthermore, although *CgSNF3* gene deletion had no effect on *CgRGT1* and *CgHXT3* gene expression, it led to diminished expression of the *CgHXT1* gene (Fig. 5*A*). In this context, it is noteworthy that Ng *et al.* (34) have previously found lower, higher, and similar transcript levels of *CgMIG1* and *CgHXT1*, *CgHXT3*, and *CgHXT2/10* and *CgRGT1* genes, respectively, in the *Cgsnf3Δ* mutant, compared with *wt* cells. The reason for this discrepancy in *CgHXT3* and *CgHXT2/10* gene expression in theirs and our data is not understood and warrants further investigation.

Intriguing, the *Cgsnf3Δyps1-11Δ* mutant displayed transcriptional profiles that were in-between the two mutants *Cgsnf3Δ* and *Cgyps1-11Δ*, with higher and lower transcript levels of *CgRGT1*, and *CgMIG1*, *CgHXT1*, *CgHXT2/10* (*I*) ,and *CgHXT3* genes, respectively, in the *Cgsnf3Δyps1-11Δ* mutant (Fig. 5*A*). Collectively, these data implicate CgSnf3 in transcriptional activation of *CgMIG1* and *CgHXT2/10* (*I*) genes, whereas *CgRGT1* and *CgHXT3* gene expression is probably not regulated by CgSnf3. Therefore, *CgRGT1* induction and *CgHXT3* repression in *Cgyps1-11Δ* and *Cgsnf3Δyps1-11Δ* mutants is likely to be dependent upon the signaling pathway regulated by other glucose sensor/s.

Since the human host niches, blood, vagina, and urine, have varied glucose levels, and sugar sensing mechanisms are pivotal to microbial growth (22, 41, 42), we next examined the response of *C. glabrata* to extracellular glucose by measuring the expression of *CgMIG1*, *CgRGT1*, *CgSNF3*, *CgHXT1*, *CgHXT2/10* (*I*), and *CgHXT3* genes in regular- (2%), low- (0.03%), and high- (5%) glucose medium–grown cells. Moreover, to determine the role of CgYapsins and CgSnf3 in glucose sensing and transport, we examined transcript levels in *Cgyps1-11Δ*, *Cgsnf3Δ*, and *Cgsnf3Δyps1-11Δ* strains as well. We observed that *wt* cells respond to glucose starvation by inducing the expression of *CgMIG1*, *CgRGT1*, *CgSNF3*, *CgHXT1*, and *CgHXT3* genes and repressing the transcription of *CgHXT2/10* (*I*) gene (Fig. 5*B*). Contrarily, growth in the surplus-glucose medium led to transcriptional downregulation and upregulation of *CgMIG1* and *CgHXT2/10* (*I*) genes, respectively, while having no effect on expression of the remainder genes (Fig. 5*B*). These data suggest a reciprocal regulation of *CgMIG1* and *CgHXT2/10* (*I*) genes based on the extracellular glucose abundance and preclude *CgRGT1*, *CgSNF3*, *CgHXT1*, and *CgHXT3* genes from cellular transcriptional response to the high-glucose environment. Of note, Ng *et al.* (33) also have previously shown upregulation of *CgMIG1*, *CgRGT1*, *CgSNF3*, and *CgHXT3* in response to low glucose. However, we noticed one difference in the low-glucose-responsive gene expression data, which was for the *CgHXT1* gene, with *CgHXT1* being upregulated and downregulated in our and their study, respectively, upon exposure to low-glucose conditions (33), which could in part be due to different growth conditions (30 °C *versus* 37 °C).

Furthermore, the *Cgyps1-11Δ* mutant could not respond to low-glucose (0.03% glucose) medium *via* transcriptional activation of *CgMIG1*, *CgRGT1*, and *CgSNF3* genes (Fig. 5*C*), although these genes had higher basal transcript levels in the mutant (Fig. 5*A*). In fact, *CgMIG1* transcript levels were lower in low-glucose–grown *Cgyps1-11Δ* cells, compared with 2% glucose–grown *Cgyps1-11Δ* cells (Fig. 5*C*). Despite having deregulated basal expression of *CgHXT2/10* (*I*) and *CgHXT3* genes, the *Cgyps1-11Δ* mutant, similar to *wt* cells, showed low-glucose–responsive upregulation and downregulation of *CgHXT3* and *CgHXT2/10* (*I*) genes, respectively (Fig. 5*C*). Of note, *CgHXT1* gene expression remained unchanged in low-glucose–grown *Cgyps1-11Δ* cells, compared with 2% glucose–grown *Cgyps1-11Δ* cells (Fig. 5*C*). Altogether, these data suggest that the loss of CgYapsins results in an impaired transcriptional response to glucose limitation (Fig. 5*C*). Furthermore, the transcriptional response of the *Cgyps1-11Δ* mutant to high glucose was dissimilar to that of the *wt* strain for *CgMIG1* and *CgHXT2/10* (*I*) genes, as *CgMIG1* and *CgHXT2/10* (*I*) genes were not downregulated and upregulated, respectively, in high-glucose–grown *Cgyps1-11Δ*, compared with 2% glucose–grown *Cgyps1-11Δ* cells (Fig. 5*C*), underscoring that the loss of CgYapsins also affects the cellular transcriptional response to surplus glucose.

The transcriptional response of the *Cgsnf3Δ* mutant to low glucose was similar to that of the *wt* strain except for the *CgHXT1* gene, which was downregulated in the mutant (Fig. 5*D*), implicating CgSnf3 in both basal and glucose-limitation-responsive activation of *CgHXT1* gene expression. However, this *wildtype*-like transcriptional response to the low-glucose environment was unexpected for the *Cgsnf3Δ* mutant, as *Cgsnf3Δ* cells could not grow well in glucose-limited conditions (Figs. 3*A* and 4*B*). This anomaly could be due to altered expression of other hexose transporters in the mutant. Alternatively, it may stem from differences in the growth conditions (continuous glucose starvation [serial dilution spotting-based growth analysis] *versus* 2-h growth in low-glucose medium [growth condition for qRT-PCR experiment]), and, thus, a systematic kinetic study of gene expression in glucose-limited conditions may shed light on these two possibilities.

The *Cgsnf3Δ* mutant also mounted *wt*-like transcriptional response to high glucose for the *CgHXT2/10* (*I*) gene, whose levels were elevated in 5% glucose–grown *Cgsnf3Δ* cells, compared with 2% glucose–grown *Cgsnf3Δ* cells (Fig. 5*D*). However, contrary to *wt* cells, *CgMIG1* gene expression remained unchanged between 2% and 5% glucose–grown *Cgsnf3Δ* cells (Fig. 5*D*). Unlike *wt* cells, the *Cgsnf3Δ* mutant displayed increased expression of the *CgHXT1* gene in response to high glucose (Fig. 5*D*). These data suggest that CgSnf3 is required for transcriptional downregulation of *CgMIG1* in the surplus-glucose environment; however, the high-glucose–induced expression of *CgHXT1* in the *Cgsnf3Δ* mutant is likely to be mediated by another transcriptional regulator.

Although the *Cgsnf3Δyps1-11Δ* mutant responded to glucose limitation by activating *CgMIG1* gene expression, it was, like the *Cgyps1-11Δ* mutant, deficient in activating *CgRGT1* (Fig. 5*E*), thereby pointing toward a CgSnf3-indepdendent role of CgYapsins in regulating *CgRGT1* gene expression. Of note, the *Cgsnf3Δyps1-11Δ* mutant exhibited low-glucose–responsive transcriptional downregulation of the *CgHXT1* gene, similar to the *Cgsnf3Δ* mutant (Fig. 5*E*). Furthermore, like in *wt*, *Cgyps1-11Δ*, and *Cgsnf3Δ* strains, *CgHXT2/10* (*I*) and *CgHXT3* transcription was downregulated and upregulated, respectively, in response to low glucose (Fig. 5*E*). The transcriptional response of the *Cgsnf3Δyps1-11Δ* mutant to high glucose was complex, and identical to neither *Cgsnf3Δ* nor *Cgyps1-11Δ* mutant for genes analyzed, and involved downregulation of all genes except for *CgHXT1* and *CgHXT3* genes, whose expression was higher and similar, respectively, between 5% and 2% glucose–grown *Cgsnf3Δyps1-11Δ* cells (Fig. 5*E*), suggesting a complex interplay between CgSnf3 and CgYapsin-dependent regulation of glucose sensing and uptake genes.

Since yeast cells are known to respond to low- and high-glucose environmental conditions by upregulation and downregulation, respectively, of the high-affinity glucose transporter genes (43, 44), we draw seven major conclusions from our qRT-PCR analysis. First, both the high-affinity, low-glucose sensor CgSnf3 and the transcriptional regulators CgMig1 and CgRgt1, are transcriptionally activated in response to glucose starvation (Fig. 5*B*). Second, CgHxt1 and CgHxt3, and CgHxt2/10 (I) are likely to represent high-affinity and low-affinity glucose transporters, respectively, with the *CgHXT2/10* (*I*) gene also showing glucose-responsive reciprocal transcriptional regulation (induction in surplus-glucose and repression in low-glucose environment) (Fig. 5*B*). Third, the transcriptional response of *C. glabrata* toward surplus environmental glucose involves downregulation of *CgMIG1* and upregulation of *CgHXT2/10* (*I*) (Fig. 5*B*). Fourth, despite CgSnf3’s requirement for growth in low-glucose medium (Figs. 3*A* and 4*B*), the *Cgsnf3Δ* mutant is not drastically impaired in mounting an appropriate transcriptional response to external glucose concentrations at early stages (2 h) of glucose starvation (Fig. 5*D*). Fifth, although *CgMIG1* expression is controlled by CgSnf3-dependent glucose sensing pathway under regular-glucose (2%) condition, the transcriptional regulation of *CgRGT1* and *CgHXT3* is independent of the CgSnf3 pathway (Fig. 5*A*). Sixth, the reduced expression of *CgMIG1* and *CgHXT2/10* (*I*) genes in the *Cgsnf3Δyps1-11Δ* mutant (Fig. 5*A*) may contribute to better growth of the *Cgsnf3Δyps1-11Δ* mutant in the 2% glucose-containing medium, as compared with the *Cgyps1-11Δ* mutant (Figs. 3*A*, 4*A* and S2). Finally, the high basal transcript levels of low-glucose–responsive genes, *CgMIG1*, *CgRGT1*, and *CgSNF3,* in the *Cgyps1-11Δ* mutant (Fig. 5, *A* and *B*) suggest that the loss of CgYapsins impairs the cellular ability to sense environmental glucose, with the high-glucose environment probably being perceived as a low-glucose environment, and that, this perturbed glucose sensing and uptake pathway adversely affects the *Cgyps1-11Δ* cell physiology.

### The Cgyps1-11Δ mutant displays elevated glucose uptake

**Figure 6:**
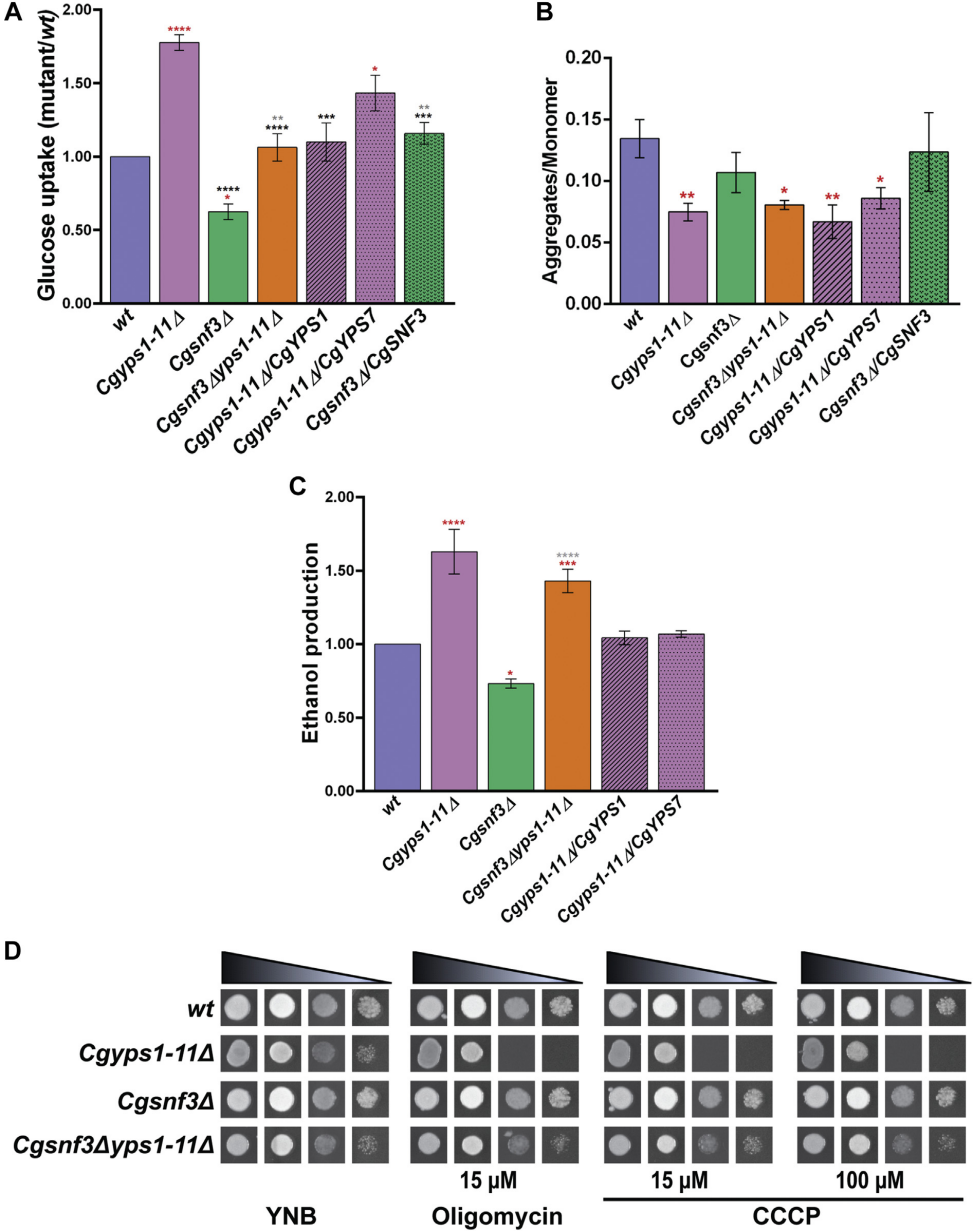
**The *Cgyps1-11Δ* mutant displays higher glucose uptake.***A*, uptake of 2-NBDG ([2-N-(7-nitrobenz-2-oxa-1,3-diazol-4-yl) amino]-2-deoxy-D-glucose) in indicated *C. glabrata* strains, as determined by spectrofluorimetry. Glucose-starved cells were incubated with 2-NBDG (100 μM) for 1 h at 30 °C, and the fluorescence emission was recorded at 540 nm, under excitation at 465 nm. Data (mean ± SEM, n = 3–5) were normalized against the *wt* fluorescence values (considered as 1.0) and represent fold change in NBDG uptake in mutant strains, compared with the *wt* strain. *Red asterisks* denote differences in the glucose uptake between *wt* and indicated strains, *black asterisks* denote differences between *Cgyps1-11Δ* and indicated strains, and *gray asterisks* denote differences between *Cgsnf3Δ* and indicated strains. ∗*p* ≤ 0.05; ∗∗*p* ≤ 0.01; ∗∗∗*p* ≤ 0.001; ∗∗∗∗*p* ≤ 0.0001, one-way ANOVA with Tukey’s test. *B*, JC-1 dye–based assessment of mitochondrial membrane potential. Log-phase cells were stained with JC-1 (20 μM) dye and washed with PBS, and fluorescence of J-aggregates (*red*) and monomers (*green*) was recorded at 550 nm excitation/emission 600 nm and excitation/emission 485 nm/535 nm, respectively. The ratio of red fluorescence (J aggregates) to green fluorescence (monomer) was calculated for each strain and plotted. Data represent mean ± SEM (n = 3–4). *Red asterisks* denote differences between *wt* and indicated strains. ∗*p* ≤ 0.05; ∗∗*p* ≤ 0.01, one-way ANOVA with Tukey’s test. *C*, ethanol measurement in the culture broth. Ethanol in the culture medium of indicated strains was extracted using dibutyl phthalate, followed by potassium dichromate oxidation of ethanol. The amount of ethanol in the culture medium was calculated from the standard curve, and data (mean ± SEM, n = 3–5) were normalized against ethanol produced by the *wt* strain (considered as 1.0). Data represent fold change in ethanol production in mutant strains, compared with the *wt* strain. *Red* and *gray asterisks* denote uptake differences between *wt* and indicated strains, and *Cgsnf3Δ* and *Cgsnf3Δyps1-11Δ* mutants, respectively. ∗*p* ≤ 0.05; ∗∗∗*p* ≤ 0.001; ∗∗∗∗*p* ≤ 0.0001, one-way ANOVA with Tukey’s test. *D*, liquid medium–based growth analysis of *wt*, *Cgyps1-11Δ*, *Cgsnf3Δ*, and *Cgsnf3Δyps1-11Δ* strains in the presence of indicated inhibitors. Cultures were inoculated at an initial *A*_600_ of 0.25 and grown in medium lacking (YNB) or containing oligomycin (15 μM) and carbonyl cyanide m-chlorophenylhydrazone (CCCP; 15 and 100 μM). After 12 h, cultures were diluted in PBS, and 3 μl of undiluted and 10-, 100-, and 1000-fold-diluted cultures were spotted on YNB medium. Plates were incubated at 30 °C, and images were captured after 1 day.

Next, to better understand the link among CgYapsins, CgSnf3, and glucose homeostasis, we performed four experiments. First, we measured glucose uptake in mutant strains using the fluorescent glucose analog 2-NBDG and found 1.8-fold higher glucose uptake in the *Cgyps1-11Δ* mutant, compared with *wt* cells (Fig. 6*A*), which is consistent with an increased expression of the high-affinity glucose sensing system in the mutant. Of note, the ectopic expression of *CgYPS1* and *CgYPS7* could fully and partially complement the elevated glucose uptake, respectively, in the *Cgyps1-11Δ* mutant (Fig. 6*A*). As a control, we also checked glucose uptake in the *Cgsnf3Δ* mutant and found it to be 1.6-fold lower than that in *wt* cells, which was restored back to *wt* levels upon ectopic expression of the *CgSNF3* gene (Fig. 6*A*). Deletion of the *CgSNF3* gene in the *Cgyps1-11Δ* mutant led to diminished glucose uptake, which was similar to that of the *wt* strain but more than that of the *Cgsnf3Δ* mutant (Fig. 6*A*), thereby implicating both CgSnf3-dependent and CgSnf3-independent pathways in controlling glucose uptake in the *Cgsnf3Δyps1-11Δ* mutant. These results are consistent with our qRT-PCR-based gene expression data (Fig. 5) and suggest that glucose uptake in *C. glabrata* is regulated by the CgSnf3 sensor and the elevated glucose uptake in *Cgyps1-11Δ* mutant is largely dependent upon CgSnf3.

Second, we explored the possibility if elevated glucose uptake in the *Cgyps1-11Δ* mutant has any effect on mitochondrial functions. For this, we checked the mitochondrial membrane potential using the lipophilic JC-1 dye, which, upon entry into the mitochondria, displays a shift from green to red fluorescence, with green and red fluorescence emitted by native/free dye molecules and J-dye aggregates, respectively, with J-aggregates forming in the energized and negatively charged mitochondria (45). JC-1 dye accumulation in the mitochondria is dependent upon the mitochondrial membrane potential, with the depolarized mitochondria with reduced membrane potential exhibiting a lower fluorescence shift from green to red (45). Therefore, the reduced red/green fluorescence ratio of JC-1 is used as an indicator of the depolarization of the mitochondrial membrane. To measure the mitochondrial membrane potential in *wt* and *Cgyps1-11Δ*, we stained log-phase cells with JC-1 dye and measured the red and green fluorescence in excitation/emission, 550 nm/600 nm and 485 nm/535 nm, respectively. We found the ratio of red to green fluorescence to be 1.8-fold lower in the *Cgyps1-11Δ* mutant, compared with the *wt* strain (Fig. 6*B*), indicating diminished JC-1 aggregate formation, probably owing to a decline in the mitochondrial membrane potential. Of note, ectopic expression of neither *CgYPS1* nor *CgYPS7* could restore the reduced red to green fluorescence ratio in the *Cgyps1-11Δ* mutant (Fig. 6*B*), underscoring the contribution of multiple CgYapsins to maintenance of the mitochondrial membrane potential. Furthermore, the *Cgsnf3Δ* mutant displayed *wt*-like JC-1 accumulation pattern, whereas the *Cgsnf3Δyps1-11Δ* mutant was defective in fluorescence shift from red to green and exhibited a 1.7-fold diminished ratio of red to green fluorescence (Fig. 6*B*). Altogether, these data suggest that, despite normal mitochondrial morphology (Fig. 2*A*), the mitochondrial membrane potential is significantly impaired in the *Cgyps1-11Δ* mutant and CgYapsins may play a role in mitochondrial homeostasis.

**Supplemental Figure S3:**
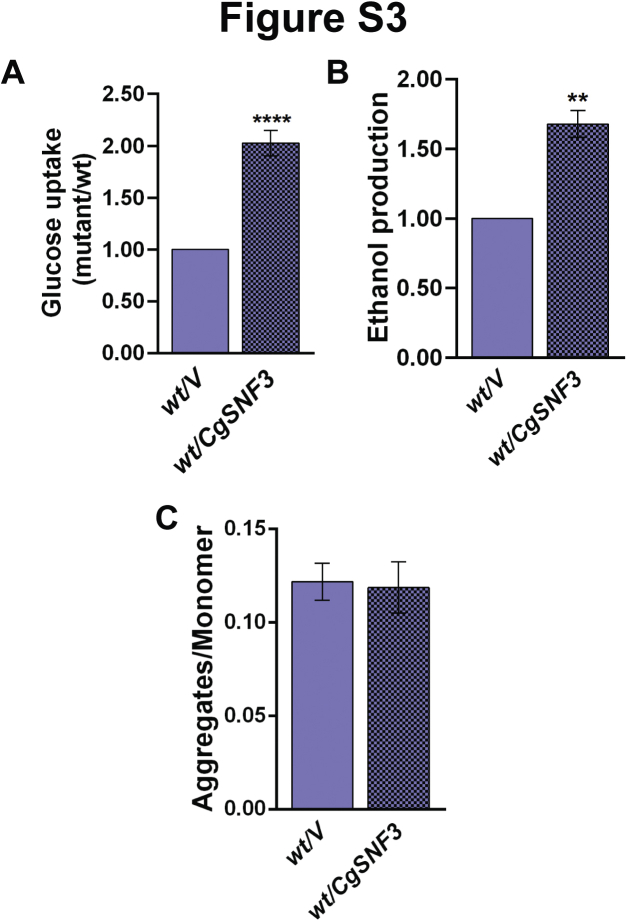


Based on the above data, we next reasoned that the defective glucose sensing pathway and the depolarized mitochondria in the *Cgyps1-11Δ* mutant may impact the cellular energy metabolic pathways, *viz.*, fermentation and respiration. To test this hypothesis, we first measured the amount of ethanol produced in the culture media of *wt* and *Cgyps1-11Δ* cells. For this, ethanol was extracted using the nonalcoholic solvent dibutyl phthalate, followed by ethanol oxidation with potassium dichromate. We found 1.6-fold higher levels of ethanol in the culture supernatant of *Cgyps1-11Δ* cells, compared with the *wt* culture medium (Fig. 6*C*), suggestive of an increased flux through glycolysis in *Cgyps1-11Δ* cells, which may lead to higher production of ethanol from the glycolysis-derived pyruvate. Intriguing, *CgYPS1* and *CgYPS7* expression could rescue increased ethanol production in *Cgyps1-11Δ* cells, whereas the *Cgsnf3Δyps1-11Δ* mutant displayed higher, higher, and similar ethanol production, compared with *wt*, *Cgsnf3Δ*, and *Cgyps1-11Δ* cells, respectively (Fig. 6*C*). *Cgsnf3Δ* mutant cells exhibited 25% less ethanol production compared with *wt* cells (Fig. 6*C*). Although this result implicates CgSnf3 in fermentative metabolism in *C. glabrata*, the similar ethanol production in *Cgyps1-11Δ* and *Cgsnf3Δyps1-11Δ* mutants indicate that *CgSNF3* is unlikely to be the sole contributor to the increased fermentative metabolism in the *Cgyps1-11Δ* mutant. To corroborate these results further, we investigated the effect of *CgSNF3* overexpression on glucose uptake, mitochondrial potential, and ethanol production in *C. glabrata*. We found that the overexpression of *CgSNF3* in *wt* cells mimicked the behavior of the *Cgyps1-11Δ* mutant for glucose uptake and ethanol production, as the *wt/CgSNF3* strain exhibited a 2-fold elevated glucose uptake (Fig. S3*A*) and 1.5-fold higher ethanol production (Fig. S3*B*), as compared with *wt* cells expressing the empty vector. Of note, *CgSNF3* overexpression had no effect on the mitochondrial membrane potential, as the ratio of red to green fluorescence of the JC-1 dye was similar between *wt* cells carrying empty vector and overexpressing *CgSNF3* (Fig. S3*C*). Together, these data suggest that CgSnf3 is a critical component of glucose uptake and homeostasis system, and higher *CgSNF3* transcript levels in the *Cgyps1-11Δ* mutant are likely to account for perturbed glucose homeostasis while mitochondrial perturbation may not be solely dependent upon CgSnf3.

Finally, since the cellular capacity to activate respiration has previously been reported to undergo a steady deterioration in the continued presence of glucose (46), and we found *Cgyps1-11Δ* cells to exhibit elevated glucose uptake (Fig. 6*A*), diminished mitochondrial membrane potential (Fig. 6*B*), and reduced ATP levels (24), we next hypothesized that the respiratory metabolism may be impaired in *Cgyps1-11Δ* cells. To test this, we examined the effect of oxidative phosphorylation inhibition on the viability of *Cgyps1-11Δ* cells. Liquid media-based growth analysis revealed an increased sensitivity of the *Cgyps1-11Δ* mutant to inhibitors oligomycin (mitochondrial ATP synthase inhibitor) and carbonyl cyanide m-chlorophenylhydrazone (CCCP; oxidative phosphorylation uncoupler) (Fig. 6*D*). The *Cgsnf3Δyps1-11Δ* mutant exhibited much better growth in the presence of oxidative phosphorylation inhibitors than the *Cgyps1-11Δ* mutant (Fig. 6*D*), suggesting that loss of the high-affinity glucose sensing system partially compensates for impaired oxidative phosphorylation in the *Cgyps1-11Δ* mutant. Altogether, our data raise the possibility that the respiratory mode of energy production may be crippled in the *Cgyps1-11Δ* mutant, which may lead to an overreliance on the fermentative metabolism for growth. Of note, a *S. cerevisiae* strain displaying reduced glucose uptake, due to the presence of a chimeric hexose transporter, has been reported to exhibit entirely respiratory metabolism even in the presence of high glucose (47). In this context, it is noteworthy that the increased and diminished abundance of glycolytic enzymes and ETP proteins, respectively, in the membrane proteome (Fig. 1, *E* and *F*) and the altered mitochondrial membrane potential (Fig. 6*B*) in *Cgyps1-11Δ* mutant also point toward the possibility of accelerated fermentative and inadequate respiratory metabolism in the mutant. However, it remains to be determined whether sensing of the glucose-rich environment as the glucose-poor environment by *Cgyps1-11Δ* cells is a cause or an effect of elevated glycolytic flux. Also, if and how glucose missensing affects the transcriptional regulation of fermentative and respiratory metabolic genes warrants further investigation.

Altogether, our data yielded four new findings. First, CgSnf3 is involved in glucose sensing, as the mutant lacking the *CgSNF3* gene showed reduced glucose uptake (Fig. 6*A*) and altered expression of genes involved in glucose sensing and transport (Fig. 5*A*). Second, the diminished abundance of hexose transporters, CgHxt1 and CgHxt4, in the membrane proteome of the *Cgyps1-11Δ* mutant (Table S11) could be due to their transcriptional downregulation in the mutant (Fig. 5*A*). Third, the glucose and mitochondria homeostasis are perturbed in the *Cgyps1-11Δ* mutant, which could in part be attributed to the impaired CgSnf3-dependent glucose sensing. Fourth, the dysfunctional mitochondria in the *Cgyps1-11Δ* mutant either emerge from or lead to inadequate respiratory metabolism during cellular growth.

In conclusion, we demonstrate unequivocally for the first time Yapsin-dependent modulation of glucose homeostasis in any pathogenic fungus, and our findings have wide applications in design of strategies to better control fungal infections and understand glucose metabolism pathways.

## Discussion

Glucose metabolism is essential for life, as it plays a central role in generation of energy and biosynthetic building blocks. Glucose homeostasis in fungi is maintained by a finely tuned system consisting of glucose sensors, transporters, and metabolic enzymes (43). Based on the environmental glucose abundance, expression of the hexose transporter–encoding genes is controlled by specific transcription factors of the extracellular glucose sensing pathways (43, 44). Since the human host niches that pathogenic fungi are exposed to vary widely in glucose levels, *viz.*, blood (2–30 mM), vaginal fluids (5–149 mM), and urine (0–0.8 mM), glucose sensing mechanisms are pivotal to fungal virulence (41, 42). Therefore, the ability of *Candida* species to sense and appropriately respond to extracellular glucose is likely to contribute to their success as both bloodstream and urinary and vaginal tract pathogens (6, 7, 8, 22).

The pathogenic yeast *C. glabrata* is known to have two plasma membrane glucose sensors, CgSnf3 and CgRgt2, which are involved in sensing glucose-deficient and glucose-surplus environmental conditions, respectively, by regulating the expression of hexose transporters through transcription factors CgRgt1 and CgMig1 in response to glucose abundance (33, 34). Although the mechanistic basis for glucose sensing in *C. glabrata* is still elusive, the glucose transport and homeostasis mechanisms are well defined in its evolutionarily close relative *S. cerevisiae* (44, 48). Glucose repression, which involves deregulation of alternate carbon source utilization genes in the presence of the preferred carbon source glucose, plays a key role in glucose metabolic regulation in *S. cerevisiae* (43, 44). Furthermore, the hexose transporters, which have varied (high, moderate, and low) affinity for glucose, primarily import glucose *via* facilitated diffusion, with the Snf3/Rgt2-Rgt1 pathway regulating their gene expression (22, 43, 44). Rgt1 in *S. cerevisiae* is known to inhibit the expression of hexose transporter–encoding genes (*HXTs*), in the presence of glucose, *via* formation of a repressor complex with Mth1, Std1, Ssn6, and Tup1 corepressors (43, 44, 48). The binding of glucose to the plasma membrane glucose sensors Rgt2 and Snf3 results in casein kinase I (Yck1/Yck2)-mediated phosphorylation of Mth1 and Std1, which leads to their proteasomal degradation, thereby preventing the binding of Rgt1 to the *HXT* gene promoters (22, 43, 44, 48). Snf3 and Rgt2 serve as high- and low-affinity glucose receptors, respectively, with Snf3 being required for low-glucose–induced transcriptional activation of moderate-affinity glucose transporters, Hxt2 and Hxt4, and Rgt2 being involved in elevating expression of the low-affinity hexose transporter, Hxt1 (22, 43, 44, 48). Another transcriptional repressor Mig1, which contains Cys2His2 zinc finger DNA-binding motif, is involved in transcriptional downregulation of its target moderate- and high-affinity hexose transporter–encoding genes when glucose is present in high abundance, along with general corepressors, Ssn1 and Tup1 (22, 43, 44, 48). Under glucose starvation conditions, Mig1 is phosphorylated by Snf1 kinase, which inhibits its repressor activity and leads to its export from the nucleus (22, 43, 44, 48). With this multilayered, tightly controlled glucose sensing and uptake system, *S. cerevisiae* is able to grow over a wide range of environmental glucose concentrations (22, 44).

*C. glabrata* possesses orthologs of *S. cerevisiae* glucose sensors, transporters, and transcriptional regulators (22, 33, 34, 35). While CgSnf3 and CgRgt2, and CgRgt1 and CgMig1 are postulated to act as glucose sensors and transcription factors, respectively, in *C. glabrata*, its 11 hexose transporters (CgHxts) also share similarity with their *S. cerevisiae* counterparts (22, 33, 34, 35). Moreover, the upregulation of *CgHXT3* and *CgHXT5* genes under low-glucose growth conditions (33) suggests that these probably are high-affinity hexose transporters. Furthermore, consistent with fungal glucose sensors possessing long (about 200 aa) cytoplasmic C-terminal tails containing a 25-amino-acid sequence motif, which is involved in extracellular glucose signaling (22, 43), CgRgt2 and CgSnf3 also carry one copy of this motif in their C-terminal tails (33). Of note, the cytoplasmic C-terminal tail is absent in fungal hexose transporters (22, 35, 43). In the current study, we have shown that CgSnf3 is required for both uptake of glucose (Fig. 6*A*) and transcriptional regulation of glucose homeostasis genes (Fig. 5*A*), and our results place CgSnf3 at the forefront of glucose transport regulatory mechanisms. Furthermore, our finding of the nonresponsiveness of *CgSNF3* gene expression to the high environmental glucose (Fig. 5*D*) is in accordance with its role primarily in sensing glucose-limited conditions (34).

**Supplemental Figure S4:**
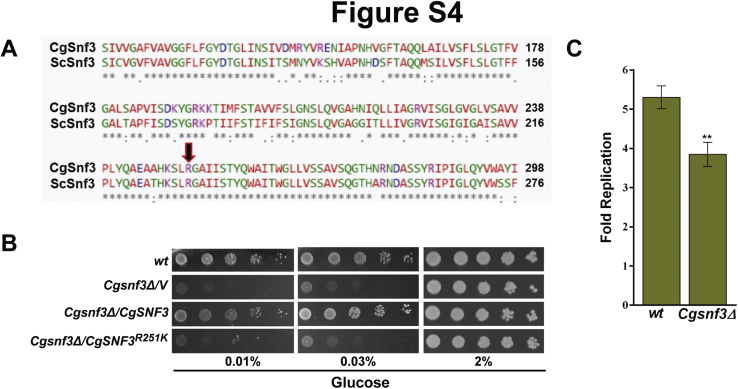


Despite the similar composition of glucose sensing pathways in *S. cerevisiae* and *C. glabrata* (22, 35), the individual pathway constituents may not function in an identical manner. For example, substitution of the conserved arginine 251 residue with lysine (R251K) in CgSnf3 led to impaired functions of CgSnf3 in survival under low environmental glucose conditions (Fig. S4, *A* and *B*), whereas the same mutation (R229K) rendered *S. cerevisiae* Snf3 to be constitutively active (49). Similarly, *CgRGT1* gene expression is regulated in response to glucose abundance in *C. glabrata* (Fig. 5*B*; (33)), contrary to its *S. cerevisiae* counterpart (50). Furthermore, our transcript profiling of glucose homeostasis genes suggests a complex regulation of glucose sensing pathways, with *CgMIG1* and *CgRGT1* transcription being activated in response to both high and low glucose, and low-glucose environment, respectively (Fig. 5*B*). Our data also suggest that CgSnf3 is likely to be the major but not the sole sensor and/or transducer of glucose-limited environment, as the *Cgsnf3Δ* mutant, despite having low basal transcription of *CgMIG1* (Fig. 5*A*), was able to activate *CgMIG1* gene expression in response to glucose starvation (Fig. 5*D*). Moreover, consistent with the previous study (34), the *Cgsnf3Δ* mutant displayed diminished intracellular replication in human THP-1 macrophages, compared with *wildtype* cells (Fig. S4*C*), with the macrophage internal milieu being a glucose-deficient environment (6). Altogether, these data raise the possibility that the extracellular sensing and homeostasis of glucose in *C. glabrata* is probably maintained by partially functionally redundant proteins. Moreover, variations in glucose metabolic pathways between *S. cerevisiae* and *C. glabrata* may have evolved owing to different habitats of these two yeasts. In this context, it is noteworthy that, unlike *S. cerevisiae*, *C. glabrata* can utilize only two sugars, glucose and trehalose, as fermentable carbon sources (22).

Macrophages play an important role in control of *Candida* infections, with glucose metabolism being central to metabolic reconfiguration of both macrophages and *Candida* cells (6, 51). Recently, it has been shown that macrophages and *C. albicans* compete for the limited amount of glucose available by turning on their glycolysis pathway (20). This activation of the glucose utilization pathway is an important determinant for the outcome of *C. albicans*–macrophage interaction, as *C. albicans* utilizes the depleted-glucose environment to trigger macrophage cell death (20). Although the role of glucose metabolism in *C. glabrata*–macrophage interaction is yet to be investigated in depth, transcriptome profiling studies have shown that *C. glabrata* induces gluconeogenesis and the glyoxylate cycle, and represses the glycolysis pathway, upon phagocytosis by macrophages (5, 6). Furthermore, genes coding for CgHxt3 and CgHxt5 hexose transporters and CgMig1 transcriptional repressor were upregulated in macrophage-internalized *C. glabrata* cells (5), which is consistent with their gene activation in a low-glucose environment under laboratory growth conditions (Fig. 5*B*) (33). Genes coding for CgHxt2/10 (D) (CAGL0D02640p/CAGL0D02662p), CgHxt4, and CgHxt6/7 (CAGL0A00737p) hexose transporters, and CgStd1 (CAGL0L10043p) transcriptional repressor, were found to be downregulated in macrophage-ingested *C. glabrata* cells, which may arise from reprogramming of the glucose metabolism in the macrophage internal milieu (5). Of note, the mutant lacking 11 CgYapsins, *Cgyps1-11Δ*, which exhibited increased basal levels of *CgHXT2/10* (*D*), *CgSNF3*, *CgRGT1*, and *CgMIG1* and decreased basal levels of *CgHXT1* and *CgHXT4* gene transcripts (Figs. 2*B* and 5*A*), has been reported to be killed, whereas *C. glabrata wildtype* cells undergo 5- to 7-fold replication in macrophages (5, 18, 19). Although the intracellular survival defect of the *Cgyps1-11Δ* mutant has been attributed to NLRP3 inflammasome activation resulting in increased IL-1β production in THP-1 macrophages (19), our data collectively raise the possibility of glucose sensing and uptake mechanisms contributing to proliferation of *C. glabrata* in macrophages.

**Supplemental Figure S5:**
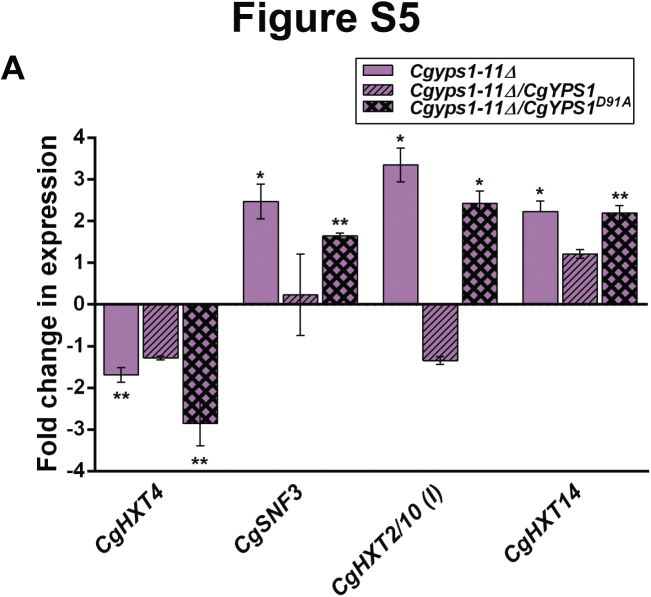


CgYapsins are known to work redundantly to ensure proper functioning of the cell wall and maintenance of vacuole and energy homeostasis (19, 24). Although *CgYPS1* expression was sufficient to rescue the defect in both intracellular survival and proliferation of the *Cgyps1-11Δ* mutant in human THP-1 macrophages, *CgYPS1* gene deletion only dampened the intracellular replication of *C. glabrata* (19), thereby pointing toward the role of other CgYapsins in interaction with macrophages. Consistently, seven *CgYPS* (*CgYPS2*, 4, 5, and 8–11) genes were upregulated in macrophage-ingested *C. glabrata* cells (5). The findings that *Cgyps1Δ* and *Cgyps7Δ* mutants do not exhibit deregulated expression of glucose sensing and uptake genes (Fig. 2*C*) and the *CgypsCΔ* (lacks nine proteases, CgYps2, 3–6, and 8–11) mutant grows like *wildtype* strain in YPD medium containing 2% glucose (5) suggest that CgYapsins are likely to perform functionally redundant roles in maintenance of glucose homeostasis. In this context, it is worth noting that GPI-anchored aspartyl proteases in *S. cerevisiae* (Yps 1–3, 6, 7) and *C. albicans* (Sap9 and 10) have not been implicated in glucose homeostasis (4, 52). Our genetic and transcriptional data suggest that glucose sensing is impaired in the *Cgyps1-11Δ* mutant, with a glucose-rich environment being perceived as a glucose-poor environment, and the dysregulated CgSnf3-dependent glucose sensing pathway partly accounts for this misperception (Figure 3, Figure 4, Figure 5, Figure 6). Although the mechanistic basis of CgYapsin-mediated regulation of glucose metabolism remains to be determined, it is possible that the glucose sensors, CgSnf3 and CgRgt2, in the plasma membrane are substrates of CgYapsins, with their processing potentially by CgYapsins generating a signal for activation of the glucose response pathway. In this context, it is worth noting that the plasma membrane flavodoxin-like protein CgPst2 has recently been shown to be a target of the CgYps1 protease (27). Furthermore, our preliminary results suggest that the proteolytic activity of CgYapsins is likely to be required for their role in glucose homeostasis, as ectopic expression of the catalytically active *CgYPS1* but not of the catalytically inactive *CgYPS1*^*D91A*^ (carries alanine in place of the catalytic residue aspartic acid and lacks proteolytic activity (27)) could restore the deregulated expression of glucose sensing and transport genes (*CgSNF3*, *CgHXT2/10* (*I*), *CgHXT4* and *CgHXT14*) in the *Cgyps1-11Δ* mutant to *wt* levels (Fig. S5). However, CgYapsin substrates involved in the erroneous glucose sensing and regulation of glucose metabolism are yet to be identified.

**Figure 7:**
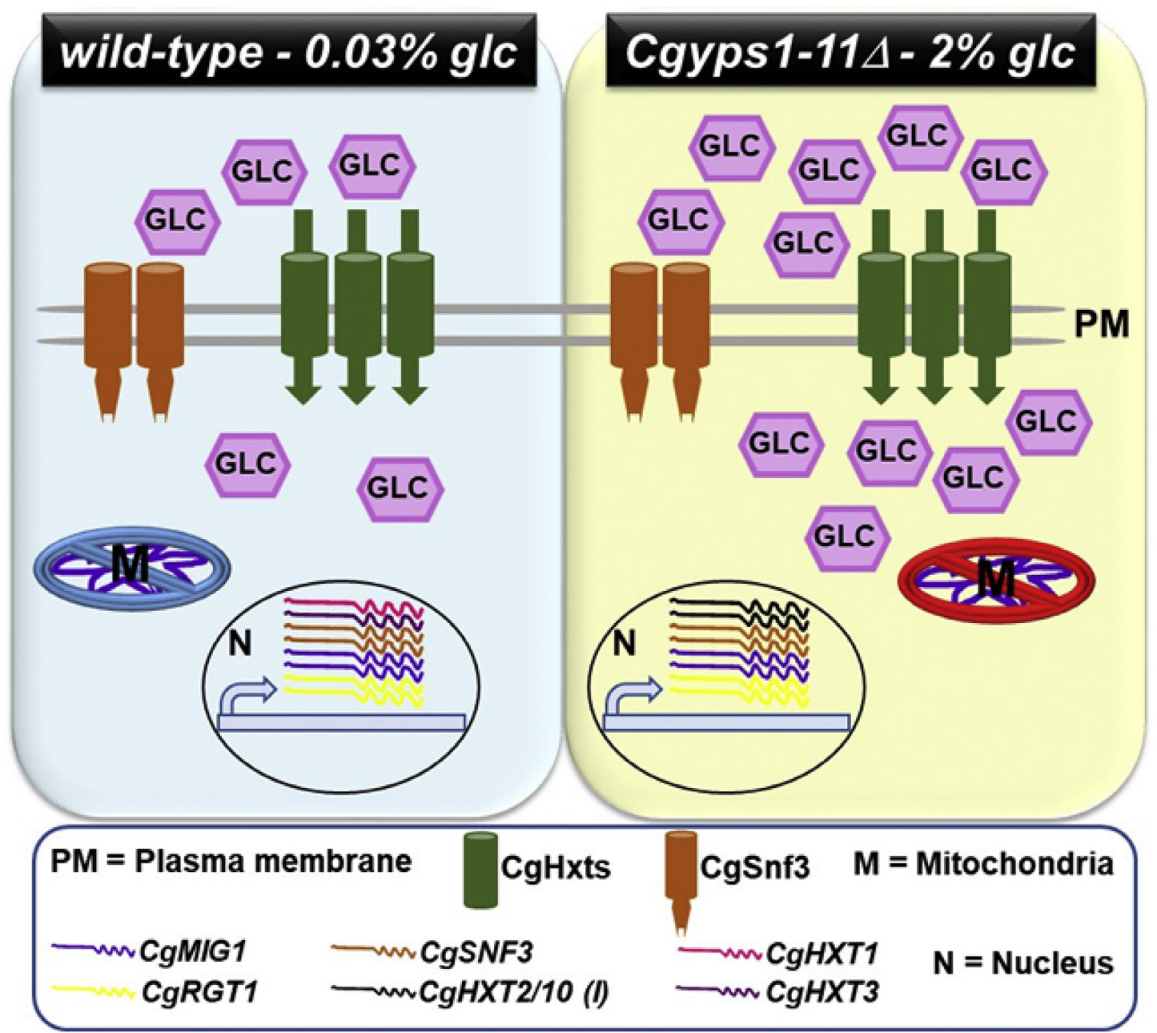
**A schematic illustration of key findings of the study.** The loss of CgYapsins impairs the ability of *C. glabrata* cells to sense the external glucose concentration. The *Cgyps1-11Δ* mutant perceives the 2% glucose environment (YPD/YNB medium) as glucose-poor environment, which results in transcriptional activation of genes coding for CgSnf3 glucose sensor, CgMig1 and CgRgt1 transcription factors, and CgHxt2/10 (I) (CAGL0I00286p) hexose transporter, which possibly leads to higher glucose uptake and perturbed glucose homeostasis. Contrarily, *wildtype* cells respond to a low-glucose environment (0.03% glucose) by elevating the expression of *CgSNF3*, *CgMIG1*, *CgRGT1*, and *CgHXT1* and *CgHXT3* (code for hexose transporters) genes, which probably facilitates glucose import, and glucose homeostasis is maintained. Of note, *CgHXT2/10* (*I*) is transcriptionally repressed in glucose-starved *wildtype* cells, whereas *CgHXT1* and *CgHXT3* genes are transcriptionally repressed in 2% glucose–grown *Cgyps1-11Δ* cells. In addition, proteins belonging to glycolysis and oxidative phosphorylation are over- and underrepresented, respectively, in total membrane proteome of the *Cgyps1-11Δ* mutant, as compared with the *wt* cells, which may contribute partly to depolarized mitochondria and elevated ethanol production in the mutant. Altogether, these data underscore a critical requirement for CgYapsins in glucose metabolism in *C. glabrata*.

In summary, we have profiled the membrane proteomes of *C. glabrata wildtype* and virulence-attenuated *Cgyps1-11Δ* mutant, performed label-free quantitative proteome analysis to explore the differential abundance of membrane proteins in these two strains, and found diminished abundance of three hexose transporters, CgHxt1, CgHxt4, and CgHxt5 in the mutant strain (Tables S11 and S19). We further showed that hexose transporters in *C. glabrata* are transcriptionally regulated in response to the environmental glucose availability and CgYapsins regulate the CgSnf3-dependent low-glucose sensing pathway as well as the mitochondrial metabolism either directly or indirectly (Fig. 7). Furthermore, the depolarized mitochondria and the increased fermentative metabolism in the *Cgyps1-11Δ* mutant, which may adversely affect both cell growth and virulence, are likely to be the physiological manifestations of high and low abundance of glycolysis and mitochondrial transport chain membrane proteins, respectively, in the mutant (Fig. 7). Since the intracellular proliferation of *C. glabrata* in macrophages is probably dependent upon an ability to switch rapidly from fermentative to respiratory metabolism, and the converse, in response to the presence of varied available carbon sources, a defect in such reprogramming may be fatal for *Cgyps1-11Δ* cells. We speculate that, in addition to energy and building block generation, remodeling of the carbon metabolism in host phagocytic cells may aid *C. glabrata* survive other host-elicited antifungal responses including reactive oxygen species production, metal ion limitation, and inflammatory responses. Of note, NLRP3 inflammasome activation has recently been shown to be incited in response to *C. albicans*-mediated glucose starvation in macrophages (21).

Altogether, our study provides an in-depth analysis of the membrane proteome of *C. glabrata* for the first time and uncovers a pivotal role for CgYapsins in glucose homeostasis. Our data suggest that CgYapsins could be a key determinant of energy (fermentative or respiratory) metabolism owing to their actions in glucose sensing mechanisms and regulating levels of mitochondrial membrane proteins.

## Experimental procedures

### Strains and growth analysis

*C. glabrata* strains used in the study (Table S20) were derived from the *C. glabrata* vaginal isolate BG2 and routinely cultured in YPD medium at 30 °C. The YPD medium contained 1% yeast extract, 2% peptone, and 2% dextrose, whereas the YNB medium contained 0.67% yeast nitrogen base and 2% dextrose. The medium containing varied glucose concentration (0.01%–5%) was prepared by adding the requisite amount of dextrose to yeast nitrogen base with ammonium sulfate for YNB medium, before autoclaving the medium. *C. glabrata* deletion strains, *Cgsnf3Δ* and *Cgsnf3Δyps1-11Δ*, were generated using the homologous recombination–based strategy with nourseothricin resistance–conferring gene *nat1* or hygromcyin resistance–conferring gene *hph1* as the selection marker (27). For generation of the *Cgsnf3Δyps1-11Δ* strain that lacks 12 genes, *CgSNF3* and *CgYPS1–11*, the *Cgyps1Δ* mutant (*CgYPS1* gene replaced *hph1* gene) was used as the parent strain and *CgSNF3* and *CgYPS2–11* genes were sequentially deleted from its genome. First, the fragment containing *CgSNF3*-5′UTR-*nat1*-*CgSNF3*-3′UTR was amplified from the genomic DNA of *Cgsnf3Δ* mutant using the Phusion High-Fidelity DNA Polymerase and transformed into the *Cgyps1Δ* strain. Transformants were selected for nourseothricin resistance, and *CgSNF3* gene deletion was confirmed by PCR. The *Cgyps1Δsnf3Δ* strain was made hygromycin sensitive by flipping out the hygromycin cassette using Flp recombinase, followed by replacement of the *CgYPS7* gene with the *hph1* gene in its genome. This step was repeated two more times, first to delete the *CgYPS2* gene, followed by replacement of the *CgYPS-Cluster* (*CgYPS3–6, 7–11*) genes with the *hph1* gene (5).

For *CgSNF3* gene cloning, the full-length *CgSNF3* ORF (2.57 kb) was amplified using the Phusion High-Fidelity DNA Polymerase, digested with restriction enzymes EcoR*I* and Sal*I*, and cloned in the pGRB2.2 plasmid under the *PGK1* promoter. For mutating arginine 251 residue to lysine in CgSnf3, mutagenic primers were used to amplify the pRK1593 plasmid containing *CgSNF3*, followed by Dpn*I* digestion and self-ligation. The replacement of the arginine codon by lysine codon in the *CgSNF3* gene was confirmed by sequencing. The oligos used in the current study are listed in Table S21.

For growth analysis in liquid medium, *C. glabrata* strains were grown in YPD medium for 12 to 16 h and inoculated in fresh YNB medium at an *A*_600_ of 0.1, followed by incubation at 30 °C with constant shaking. The culture aliquot was taken out at regular time intervals and absorbance at 600 nm was recorded. The *A*_600_ values were plotted against time, and the doubling time of each strain during logarithmic (log) phase was calculated from the slope of the curve.

### Total membrane fraction isolation

For identification of the membrane proteome, the total membrane fraction was isolated from two biological replicate samples, as described (53). Briefly, *wt* and *Cgyps1-11Δ* strains were grown overnight in the YPD medium and inoculated at an initial cell density corresponding to an *A*_600_ of 0.1 into the fresh YPD medium. After 4 h growth at 30 °C, cells were harvested, washed twice using ice-cold water, normalized to 20 *A*_600_, and suspended in 0.1 M Tris (pH 10.7), 5 mM EDTA, 2 mM dithiothreitol (DTT), and 1× protease inhibitor cocktail solution. Cells were lysed using 0.5-mm acid-washed glass beads, and cell homogenates were diluted in buffer containing 0.1 M Tris-HCl (pH 8.0), 0.33 M sucrose, 5 mM EDTA, and 2 mM DTT, followed by centrifugation at 1000*g* for 3 min at 4 °C. The supernatant was centrifuged again at 3000*g* for 5 min at 4 °C to remove unbroken cells and cell debris, if any. Next, the supernatant was centrifuged at 19,000*g* for 45 min at 4 °C to obtain the total membrane fraction pellets. The pellets were washed with the wash buffer (0.1 M Tris-HCl [pH 8.0], 0.33 M sucrose, 5 mM EDTA, and 2 mM DTT) and suspended in membrane suspension buffer containing 20% glycerol (v/v), 0.1 mM EDTA, 0.1 mM DTT, and 10 mM Tris-HCl (pH 7.5), followed by protein quantification using the BCA protein assay kit.

### Mass spectrometry analysis

For global proteome analysis of total membrane fractions, the membrane fractions (200 μg) were run on a 10% SDS-PAGE, till the bromophenol blue dye front reached 1 cm into the resolving gel. The gel was stained with Coomassie Brilliant Blue, and gel pieces containing proteins were cut and sent to Taplin Biological Mass Spectrometry Facility, Harvard Medical School, Boston, USA, for protein identification using LC-MS/MS (liquid chromatography-mass spectrometry) analysis. At the Taplin facility, proteins were digested with trypsin using in-gel digestion at 37 °C and analyzed using the Orbitrap mass spectrometer. The acquired peptide fragmentation data were analyzed using the Sequest software, run against the UniProt *C. glabrata* reference database, and identified peptides were filtered to 1% false discovery rate, as described (26).

The label-free quantitative mass spectrometry analysis of membrane proteomes was conducted at Valerian Chem Pvt Ltd, New Delhi, India. Total membrane fractions of *wt* and *Cgyps1-11Δ* strains were prepared as mentioned above, with one change in the membrane pellet suspension buffer, which contained 6 M Gn-HCL and 0.1 M Tris (pH 8.8). These samples, in duplicates, were sent on dry ice to Valerian Chem Pvt Ltd for label-free relative protein quantification, following LC-MS, using the Minora Feature Detector Node of the Proteome Discoverer 2.2 with default settings. At Valerian Chem, protein samples were reduced with TCEP solution, followed by trypsin digestion and resolution of the peptide mixture (1.0 μg) on a 25-cm-long PicoFrit column (360 μm outer diameter, 75 μm inner diameter, 10 μm tip) containing 1.8 μm of C18 resin, as described (26). The peptides were eluted with a 0% to 40% gradient of buffer containing 95% acetonitrile and 0.1% formic acid at a flow rate of 300 nl/min for 100 min. The sample analysis was carried out using the EASY-nLC 1000 system (Thermo Scientific) coupled to the QExactive mass spectrometer (Thermo Scientific) equipped with nanoelectrospray ion source, and the data-dependent top10 method was used to acquire MS data. The raw data were analyzed using the Uniprot *C. glabrata* reference database, with precursor and fragment mass tolerances set at 10 ppm and 0.5 Da, respectively, for Sequest search. The peptide spectrum match and the protein false discovery rate were set to 0.01, and the peptide spectrum matches with high and medium confidence were selected. The log2 ratio of ≥0.6 (≥1.5-fold change) for protein abundance in the *Cgyps1-11Δ* mutant to that in the *wt* strain was used to identify proteins that showed increased or decreased abundance in the mutant.

Of note, two biological replicates per strain were used for mass spectrometry experiments; the experimental details of the mass spectrometry runs and analysis are provided in Supporting Experimental Procedures, and the mass spectrometry search parameters for global and quantitative membrane proteome analysis are listed in Tables S22 and S23, respectively.

### Microscopy analysis

The MitoTracker Green dye was used to study the mitochondrial architecture as its localization to the mitochondria is independent of the mitochondrial membrane potential. YNB medium–grown log-phase cells (1.0 *A*_600_) of *wt* and *Cgyps1-11Δ* strains were collected and suspended in Hepes buffer (10 mM Hepes [pH 7.4] and 5% glucose) containing 100 nM MitoTracker Green. After 30 min incubation at room temperature in the dark, cells were washed with PBS, suspended in the Hepes buffer without MitoTracker Green, and visualized using Leica TCS SP8 confocal microscope (63/1.52NA objective). For mitochondrial morphology analysis, Z-stack images were acquired throughout the cell at 0.5-μM intervals. A set of 11 and 10 images for *wt* and *Cgyps1-11Δ*, respectively, of the entire Z-stack were projected into a single image of maximum intensity using the Leica LAS-X software.

For JC-1 staining, YPD medium–grown log-phase cells (1.0 *A*_600_) were stained with JC-1 dye (20 μM) for 10 min at 30 °C. After three washes with PBS, cells were suspended in PBS and transferred to a 96-well black opaque bottom plate. The fluorescence values of J-aggregates and monomers were recorded by excitation/emission at 550 nm/600 nm and 485 nm/535 nm, respectively. The fluorescence intensity values of unstained cells were subtracted from those of stained cells, and the ratio of red to green fluorescence was determined for each strain.

### Quantitative reverse transcription PCR

For glucose-responsive gene expression analysis, *C. glabrata* strains were grown in YNB medium for 4 h to log phase and cells were collected. After washes with PBS, cells were inoculated in the fresh YNB medium containing 0.03%, 2%, or 5% dextrose at an *A*_600_ of 1.0. After 2 h growth at 30 °C in an incubator shaker, cells were harvested and washed with chilled diethyl dicarbonate (DEPC)-treated water. Total RNA was isolated using the acid phenol extraction method and digested with DNase I. The DNase I treatment was given to eliminate residual DNA, if any. The reverse transcriptase (SuperScript III First-Strand Synthesis System for RT-PCR) was used to synthesize cDNA from DNase I-digested RNA (500 ng), followed by quantitative PCR using the SYBER Green qPCR Master Mix with appropriate set of primers. *CgACT1* gene expression was used to normalize the expression of glucose sensing and transport genes. Differences in gene expression were calculated by the comparative C_T_ (2^−ΔΔCT^) method, using two types of comparison, either 2% dextrose–grown mutant *versus* 2% dextrose–grown *wt* cells or 2% dextrose–grown *versus* 0.03%/5% dextrose–grown cells of a *C. glabrata* strain. A fold change of ≥1.5 was employed as a cutoff criterion to identify differentially expressed genes in different strains or varied growth conditions.

### BALB/c mice infection assay

For virulence analysis, 6- to 8-week-old female BALB/c mice were infected with *C. glabrata* strains. Briefly, overnight YPD medium–grown *C. glabrata* cultures were harvested, washed twice with PBS, and suspended in PBS at an *A*_600_ of 20. A volume of 100 μl cell suspension (4 × 10^7^ cells) was injected into tail veins of the BALB/c mice, and mice were monitored for 7 days. At seventh day post infection, mice were euthanized by carbon dioxide inhalation and three organs, kidneys, liver, and spleen, were collected. Organs were homogenized in sterile PBS, and appropriate dilutions were plated on the penicillin- and streptomycin-containing YPD medium. Mice infection procedures were approved by the Institutional Animal Ethics Committee (EAF/RK/CDFD/22) and were conducted at the Experimental Animal Facility of Centre for DNA Fingerprinting and Diagnostics, Hyderabad, India.

### Glucose uptake assay

*C. glabrata* strains were grown overnight in YPD medium and *A*_600_ was recorded. Cultures were inoculated in the glucose starvation medium (no-glucose YNB medium supplemented with 5% glycerol and 2% ethanol) (54) at an initial *A*_600_ of 0.1 to 0.15. After incubating for about 48 h at 30 °C, and once the *A*_600_ reached ∼1.5, cells of each strain were harvested, washed with milliQ water, and suspended in 100 μl YNB medium (without dextrose) in two sets. 2-NBDG ([2-N-(7-nitrobenz-2-oxa-1,3-diazol-4-yl) amino]-2-deoxy-D-glucose; 100 μM) was added to one set, and the other set was left as such to be used as the autofluorescence control. The cells were incubated at 30 °C for 1 h, washed twice with milliQ water, and suspended in milliQ water. Cell suspensions were transferred to a black 96-well plate, and fluorescence emission was recorded at 540 nm, under excitation at 465 nm. The relative glucose uptake was calculated by dividing the fluorescence intensity values of mutants/complemented strains by fluorescence intensity values of the *wt* strain (taken as 1.0).

### Ethanol measurement

*C. glabrata* strains were inoculated in YPD medium at an *A*_600_ of 0.3 and grown for about 4 h, till the *A*_600_ reached between 2.2 and 2.8 for each strain. Cultures were spun down, and the supernatant (3 ml) was transferred to a 15-ml tube containing dibutyl phthalate (3 ml). After vigorous vortex mixing (five times, 30 s each), tubes were centrifuged at 5000 rpm for 5 min, and 2.5 ml of the lower phase was collected. After adding 2.5 ml of potassium dichromate (0.37 M; prepared in 5 M H_2_SO_4_), tubes were vortexed vigorously (three times, 30 s each), followed by centrifugation at 5000 rpm for 5 min and collection of the lower phase (200 μl) in a 96-well plate. The plate was incubated at 37 °C for 20 min, and the absorbance was recorded at 595 nm. The amount of ethanol in the culture medium was calculated from the standard curve, prepared by using a range (0.1%–10%) of ethanol concentrations in YPD medium.

### Gene Ontology analysis

Accession numbers of proteins, identified by mass spectrometry analysis, were used to retrieve the corresponding *C. glabrata* systematic ORFs (CAGL IDs) from the UniProt Database (https://www.uniprot.org/). The CAGL IDs were used to retrieve the *S. cerevisiae* orthologs and *C. glabrata* gene description from the *Candida* Genome Database (http://www.candidagenome.org/) using the “Batch Download” option. The *S. cerevisiae* systematic ORFs and gene description were downloaded from the Yeastract repository. (http://www.yeastract.com/). The DeepLoc1.0 (http://www.cbs.dtu.dk/services/DeepLoc-1.0/index.php) server with default settings was used to determine the intracellular localization of identified proteins. Venn diagrams were prepared using the Lucid Chart (www.lucidchart.com) server. The membrane protein prediction and functional enrichment analysis was performed using the GO Slim Mapper of the *Candida* Genome Database (CGD) (http://www.candidagenome.org/cgi-bin/GO/goTermMapper), and FungiFun (https://elbe.hki-jena.de/fungifun/) and DAVID tools (https://david.ncifcrf.gov/tools.jsp), respectively.

### Other procedures

The serial dilution spotting assay and human THP-1 macrophage infection assay was performed as described (18, 27).

### Statistical analysis

The Student’s *t* test, one-way analysis of variance (ANOVA) with uncorrected Fisher’s least significant difference (LSD) test, one-way ANOVA with Tukey’s test, and Mann-Whitney U test were used for data analysis, using the GraphPad Prism software, and *p* value ≤0.05 was considered statistically significant.

## Data availability

The global and quantitative membrane mass spectrometry proteomics data have been deposited to the ProteomeXchange Consortium *via* the PRIDE partner repository with the dataset identifiers PXD029614 and PXD029629, respectively.

## Supporting information

This article contains supporting information (5, 55, 56, 57, 58, 59, 60).

## Conflict of interest

The authors declare that they have no conflict of interest with the contents of this article.

## Supporting information

Supporting Experimental Procedures, Figures S1–S5 and Tables S1–S23 Legends
