# Supplementary material for: The yapsin family of aspartyl proteases regulate glucose homeostasis in Candida glabrata
Source: J Biol Chem. 2022 Jan 17;298(2):101593. doi: 10.1016/j.jbc.2022.101593 (PMC8844688; doi:10.1016/j.jbc.2022.101593)
Supplement: Supporting Experimental Procedures, Figures S1–S5 and Tables S1–S23 Legends [file mmc1.docx]

**The yapsin family of aspartyl proteases regulate glucose homeostasis in *Candida glabrata***

Fizza Askari^#,1,2^, Mubashshir Rasheed^#,1,3^, Rupinder Kaur^1,*^

^#^Both authors contributed equally to this work.

^1^Laboratory of Fungal Pathogenesis, Centre for DNA Fingerprinting and Diagnostics, Hyderabad-500039, India

^2^Graduate studies, Regional Centre for Biotechnology, Faridabad-121001, Haryana, India

^3^Present address: MRC Centre for Medical Mycology, University of Exeter, Geoffrey Pope Building, Stocker Road, Exeter EX4 4QD, UK

^*^For correspondence: Rupinder Kaur, Centre for DNA Fingerprinting and Diagnostics (CDFD), Hyderabad, Telangana, India. Tel.: 91-40-27216137; Fax: 91-40-27216006; E-mail: [rkaur@cdfd.org.in](mailto:rkaur@cdfd.org.in).

**Supporting Information**

**Supporting experimental procedures**

**Supporting figures: S1-S5**

**Supporting tables: S1-S23**

**Supporting experimental procedures**

**Experimental details of the mass spectrometry runs and analysis**

For mass spectrometry experiments, two biological replicates per strain were used. The total membrane proteome analysis was performed at Taplin Facility, and one sample set, consisting of two biological replicates of each strain, *wild-type* and *Cgyps1-11Δ*, was used for this analysis. For quantitative total membrane proteome analysis at Valerian Chem Pvt Ltd (VProteomics), another sample set, consisting of two biological replicates of each strain, *wild-type* and *Cgyps1-11Δ*, was used.

**Total membrane proteome analysis**

For global proteome analysis of total membrane fractions of *wild-type* and *Cgyps1-11Δ* strains at Taplin Facility, Harvard Medical School, Boston, MA, USA, the excised gel bands were cut into approximately 1 mm^3^ pieces, followed by in-gel trypsin digestion using a modified procedure (55). In brief, the gel pieces were washed and dehydrated with acetonitrile for 10 min, followed by removal of acetonitrile. After complete drying of gel pieces in a speed-vac, the gel pieces were rehydrated with 50 mM ammonium bicarbonate solution containing 12.5 ng/µl modified sequencing-grade trypsin at 4ºC for 45 min. After removal of the excess trypsin solution, 50 mM ammonium bicarbonate solution was added in volume enough to just cover the gel pieces. The samples were incubated at 37ºC overnight, and peptides were extracted by removing the ammonium bicarbonate solution. After one wash with solution containing 50% acetonitrile and 1% formic acid, the samples were dried in a speed-vac for about 1 h, and stored at 4ºC until analysis.

For analysis, the samples were reconstituted in 5 - 10 µl of HPLC solvent A (2.5% acetonitrile, 0.1% formic acid), and loaded, via a Famos auto sampler, on the nano-scale reverse-phase HPLC capillary column (100 µm inner diameter x ~30 cm length) that was packed with 2.6 µm C18 spherical silica beads with a flame-drawn tip (56). A gradient was formed and peptides were eluted with increasing concentrations of solvent B (97.5% acetonitrile, 0.1% formic acid). The eluted peptides were subjected to electrospray ionization and analysed using an LTQ Orbitrap Velos Pro ion-trap mass spectrometer (Thermo Fisher Scientific, Waltham, MA). The isolation width to isolate the precursor ions was set at 2 m/z. The MS1 scan was acquired at 60,000 resolution, and the MS2 was acquired at low resolution, with fragmentation done in the ion-trap. The peptides were detected, isolated, and fragmented to produce a tandem mass spectrum of specific fragment ions for each peptide. The data-dependent top 20 method, with dynamic exclusion on, was used to acquire data. The Sequest software (57) was used to determine the peptide sequences and the protein identity by matching the UniProt *C. glabrata* reference database with the acquired fragmentation pattern. All four samples were analysed individually, and proteins, that were identified in both biological replicate samples, and represented by a minimum of two total peptides in each sample, were selected for further analysis.

**Label-free quantitative membrane proteome profiling by LC-MS Analysis**

For label-free quantitative proteome analysis of total membrane fractions of *wild-type* and *Cgyps1-11Δ* strains at Valerian Chem Pvt. Ltd., New Delhi, India, the trypsin-digested samples (1.0 µg) were resolved on a 25-cm long PicoFrit column (360 µm outer diameter, 75 µm inner diameter and 10 µm tip) filled with 1.9 µm-C18 resin on nano1000 chromatography system (Proxeon, Thermo) attached to QExactive mass spectrometer. The peptides were loaded with buffer A and eluted with a 0-40% gradient of buffer B (95% acetonitrile, 0.1% formic acid) at a flow rate of 300 nl/min for 100 min. The QExactive spray voltage was set at 2.5 kV, S lens RF level at 50 and ITC heated capillary temperature at 275°C. The MS data were acquired in positive polarity using a data-dependent method choosing 10 most intense peaks with charge state +2 to +5, with exclude isotope option enabled and dynamic exclusion of time of 12 sec. The MS1 (mass range 150-2000 m/z) and MS2 scans were acquired in Orbitrap Mass analyzer with resolution of 70,000 and 17,500 at m/z 200, respectively, with lockmass (445.12003) option enabled.

The MS1 or Full scan target was 1×106 with a maximum fill time of 100 ms with mass range set to 350−1700. The target value for MS2 or fragment scans was set at 1×105, and the intensity threshold was set at 5×103. The isolation window for the parent ion of interest was set at 2 m/z. The normalized collision energy for Higher-energy collisional dissociation (HCD) was set at 27, and the peptide match option was set to the preferred mode along with activation of the isotope exclusion option.

All samples were processed and 4 raw files generated were analyzed with the Proteome Discoverer (v2.2) against the Uniprot *C. glabrata* reference proteome database. For Sequest search, the precursor and fragment mass tolerances were set at 10 ppm and 0.5 Da, respectively. For quantification measurements, the abundance values were grouped together for two biological replicates of each strain. The proteins, that were identified with high (<0.01 FDR) or medium (<0.05 FDR) confidence, were considered for further analysis. The proteins, whose abundance was altered by ≥1.5-fold in the *Cgyps1-11Δ* mutant, as compared to the *wild-type* strain, were categorized as differentially abundant.

**Supporting figures: S1-S5**

**Figure S1: Heat map depicting abundance of proteins identified by quantitative membrane proteome profiling, that belong to carbon metabolism.** Please note that *CAGL0C03223g*, *CAGL0E03850g*, *CAGL0G09383g* and *CAGL0J00451g* are labelled as Sdh2_1 and Sdh2_2, Tdh3_1 and Tdh3_2, respectively.

**Figure S2: Growth curve analysis of *wt, Cgyps1-11Δ*, *Cgsnf3Δ* and *Cgsnf3Δyps1-11Δ* strains in YPD medium.** *C. glabrata* strains were grown overnight in YPD medium, and inoculated in fresh YPD medium at an initial OD_600_ of 0.1. Cultures were incubated at 30˚C with continuous shaking (200 rpm), and absorbance was monitored at regular intervals till 48 h. The absorbance (OD_600_) values are plotted against time, and the growth period, that corresponds to log-phase (between 2 and 6 h), was used to determine the doubling time. Data represent mean ± SEM (n = 3). The one-way ANOVA with post-hoc Tukey’s test was employed to determine the statistical significance of doubling time differences between strains. Red and black asterisks denote differences in doubling time between *wt* and *Cgyps1-11Δ* mutant, and *Cgyps1-11Δ* and *Cgsnf3Δyps1-11Δ* mutants, respectively. ****, p≤0.0001.

**Figure S3: *CgSNF3* overexpression results in elevated glucose uptake and ethanol production in *wild-type* cells.**

1. Spectrofluorimetry-based uptake analysis of 2-NBDG [2-N-(7-nitrobenz-2-oxa-1,3-diazol-4-yl) amino]-2-deoxy-D-glucose in the *wt* strain expressing either vector (*wt/V*) or *CgSNF3* (*wt/CgSNF3*). Glucose-starved cells were incubated with 2-NBDG (100 µM) for 1 h at 30˚C, and the fluorescence emission was recorded at 540 nm, under excitation at 465 nm. Data (mean ± SEM, n = 3) were normalized against *wt/V* fluorescence values (considered as 1.0), and represent fold change in NBDG uptake in the *wt/CgSNF3* strain, compared to the *wt/V* strain. ****, p≤0.0001, paired two-tailed Student's t-test.
2. Ethanol measurement. Ethanol in the culture medium of the *wt* strain expressing either vector (*wt/V*) or *CgSNF3* (*wt/CgSNF3*) was extracted using DBP, followed by potassium dichromate oxidation of ethanol. The amount of ethanol in the culture medium was calculated from the standard curve, and data (mean ± SEM, n = 4) were normalized against ethanol produced by the *wt/V* strain (considered as 1.0). Data represent fold change in ethanol production in the *wt/CgSNF3* strain, compared to the *wt/V* strain. **, p≤0.01, paired two-tailed Student’s t-test.
3. Mitochondrial membrane potential assessment using the JC-1 dye. Log-phase cells of the *wt* strain expressing either vector (*wt/V*) or *CgSNF3* (*wt/CgSNF3*) were stained with JC-1 (20 µM) dye, washed with PBS, and fluorescence of J-aggregates (red) and monomers (green) was recorded at 550 nm/emission 600 nm and excitation/emission 485 nm/535 nm, respectively. The ratio of red fluorescence (J aggregates) to green fluorescence (monomer) was calculated for both strains, and plotted. Data represent mean ± SEM (n = 4).

**Figure S4: CgSnf3 carrying mutation of the conserved arginine-251 residue to lysine is non-functional.**

1. Pair-wise sequence alignment of *C. glabrata* Snf3 [CgSnf3; CAGL0J09020g (856 aa long)] and *S. cerevisiae* Snf3 [ScSnf3; YDL194W (884 aa long)] proteins displaying conserved arginine-251 residue in CgSnf3. Amino acid sequence of *C. glabrata* and *S. cerevisiae* Snf3 proteins was retrieved from CGD (http://www.candidagenome.org/) and UniProt databases (<https://www.uniprot.org/uniprot/>), respectively, and aligned and coloured using the Clustal Omega server tool (https://www.ebi.ac.uk/Tools/msa/clustalo/). Sequence alignment, corresponding to 97-298 amino acids, inCgSnf3 is shown. The arrow points towards the conserved R174 residue in CgSnf3.
2. Serial dilution spotting analysis of *Cgsnf3Δ* mutant expressing either full length *CgSNF3* or *CgSNF3* carrying lysine substitution of arginine-251 residue (*CgSNF3^R251K^*). *C. glabrata* cultures were grown overnight in YPD medium, normalized to OD_600_ of 1.0. After diluting cultures 10-fold serially in PBS, 3 μl was spotted on YNB medium containing indicated glucose concentrations. Plates were incubated at 30^ο^C, and images were captured after 1-2 days. V denotes vector.
3. Intracellular proliferation of *wt* and *Cgsnf3Δ* mutant in human THP-1 macrophages, as determined by CFU-based assay. The human THP-1 monocytes were treated with phorbol 12-myristate 13-acetate (PMA; 16 nM) for 12 h, followed by recovery in fresh RPMI medium for 12-14 h. Next, these PMA-differentiated THP-1 macrophages were infected with YPD-medium grown overnight cultures of *wt* and *Cgsnf3Δ* strains at a MoI (multiplicity of infection) of 1:10. After 2 h incubation, the non-phagocytosed *C. glabrata* cells were washed off, and the infection was continued for another 22 h. At 2 and 24 h post infection, *C. glabrata* -infected macrophages were lysed in water, followed by plating of appropriate lysate dilutions on YPD medium. After 1-2 day incubation at 30^ο^C, yeast colonies that appeared on YPD plates were counted, and fold replication for each strain was calculated by dividing the number of intracellular *C. glabrata* cells obtained at 24 h by the number obtained at 2 h. Data represent mean ± SEM (n = 5-6). **, p≤0.01, unpaired two-tailed Student's t-test.

**Figure S5: The protease activity of CgYps1 is required for its role in regulating expression of glucose sensing and transport genes**. qRT-PCR-based expression analysis of indicated genes in YNB-medium-grown log-phase *wt*, *Cgyps1-11Δ* and *Cgyps1-11Δ* expressing either *CgYPS1* or *CgYPS1^D91A^*. Data (mean ± SEM, n = 3-4) were normalized against *CgACT1* mRNA control, and represent fold change in expression in indicated strains, as compared to the *wt* strain (considered as 1.0). *, p≤0.05; **, p≤0.01, paired two-tailed Student's t-test.

**Supporting tables: S1-S23**

**Table S1:** A list of 982 proteins identified in the membrane proteome of the *wild-type* strain in global membrane proteome analysis.

**Table S2:** A list of 1096 proteins identified in the membrane proteome of the *Cgyps1-11Δ* mutant in global membrane proteome analysis.

**Table S3:** A list of 300 and 385 proteins identified in global membrane proteomes of *wild-type* and *Cgyps1-11Δ* strains, respectively, that are predicted to be membrane proteins.

**Table S4:** Enriched GO terms for biological process (BP), cellular component (CC) and molecular function (MF) categories for the *wild-type* membrane proteome, as determined by the FungiFun tool.

**Table S5:** Enriched GO terms for biological process (BP), cellular component (CC) and molecular function (MF) categories for the *Cgyps1-11Δ* membrane proteome, as determined by the FungiFun tool.

**Table S6:** A summary of DeepLoc 1.0 server-based subcellular localization analysis of proteins identified in membrane proteomes of *wild-type* and *Cgyps1-11Δ* strains.

**Table S7:** A list of 361 and 407 proteins that were identified in membrane proteomes of *wild-type* and *Cgyps1-11Δ* strains, respectively, and were present in the membrane proteome of *C. glabrata* strain, ATCC 66032.

**Table S8:** Enriched GO terms for biological process (BP), cellular component (CC) and molecular function (MF) categories for proteins that are unique to either *wild-type* (A) or *Cgyps1-11Δ* (B) membrane proteome, as determined by the DAVID tool.

**Table S9:** A list of 1084 proteins identified in the *wild-type* and/or *Cgyps1-11Δ* membrane proteome by label-free quantitative membrane proteome profiling.

**Table S10:** Enriched GO terms for biological process (BP), cellular component (CC) and molecular function (MF) categories for 1084 identified membrane proteins, as determined by the FungiFun tool.

**Table S11:** A list of 262 proteins that displayed decreased abundance in the *Cgyps1-11Δ* membrane proteome, as determined by the label-free quantitative membrane proteome profiling.

**Table S12:** A list of 189 proteins that displayed increased abundance in the *Cgyps1-11Δ* membrane proteome, as determined by the label-free quantitative membrane proteome profiling.

**Table S13:** A list of 624 proteins whose abundance was not altered between *wild-type* and *Cgyps1-11Δ* membrane proteomes, as determined by the label-free quantitative membrane proteome profiling.

**Table S14:** Enriched GO terms for biological process (BP), cellular component (CC) and molecular function (MF) categories, as determined by the FungiFun tool, in the set of 262 (**A**) and 189 (**B**) proteins, that displayed decreased and increased abundance, respectively, in the *Cgyps1-11Δ* membrane proteome.

**Table S15:** Enriched GO terms for biological process (BP), cellular component (CC) and molecular function (MF) categories, as determined by the DAVID tool, in the set of 262 (**A**) and 189 (B) proteins, that displayed decreased and increased abundance, respectively, in the *Cgyps1-11Δ* membrane proteome.

**Table S16:** A list of 709 proteins in the *wild-type* membrane proteome that were identified by both global and quantitative membrane proteome profiling.

**Table S17:** A list of 725 proteins in the *Cgyps1-11Δ* membrane proteome that were identified by both global and quantitative membrane proteome profiling.

**Table S18:** A list of 660 common proteins between *wild-type* and *Cgyps1-11Δ* membrane proteomes that were identified by both global and quantitative membrane proteome profiling.

**Table S19:** A list of *C. glabrata* genes that code for putative glucose sensors, hexose transporters and transcriptional regulators

**Table S20:** A list of strains and plasmids used in the study

**Table S21:** A list of oligonucleotide primers used in the study

**Table S22:** A list of mass spectrometry parameters used for global membrane proteome analysis

**Table S23:** A list of mass spectrometry parameters used for label-free quantitative membrane proteome profiling
